# Advances in health-promoting effects of natural polysaccharides: Regulation on Nrf2 antioxidant pathway

**DOI:** 10.3389/fnut.2023.1102146

**Published:** 2023-02-16

**Authors:** Jiang-Hong Luo, Jing Li, Zi-Chun Shen, Xiao-Fan Lin, Ao-Qiu Chen, Yi-Fei Wang, Er-Sheng Gong, Dan Liu, Qi Zou, Xiao-Yin Wang

**Affiliations:** ^1^School of Public Health and Health Management, Gannan Medical University, Ganzhou, China; ^2^Key Laboratory of Environment and Health of Ganzhou, Gannan Medical University, Ganzhou, China; ^3^Key Laboratory of Pollution Exposure and Health Intervention of Zhejiang, College of Biology and Environmental Engineering, Zhejiang Shuren University, Hangzhou, China; ^4^State Key Laboratory of Food Science and Technology, Nanchang University, Nanchang, China

**Keywords:** natural polysaccharides, Nrf2 antioxidant pathway, structural features, regulatory effects, structure-activity relationship, health-promoting

## Abstract

Natural polysaccharides (NPs) possess numerous health-promoting effects, such as liver protection, kidney protection, lung protection, neuroprotection, cardioprotection, gastrointestinal protection, anti-oxidation, anti-diabetic, and anti-aging. Nuclear factor erythroid 2-related factor 2 (Nrf2) antioxidant pathway is an important endogenous antioxidant pathway, which plays crucial roles in maintaining human health as its protection against oxidative stress. Accumulating evidence suggested that Nrf2 antioxidant pathway might be one of key regulatory targets for the health-promoting effects of NPs. However, the information concerning regulation of NPs on Nrf2 antioxidant pathway is scattered, and NPs show different regulatory behaviors in their different health-promoting processes. Therefore, in this article, structural features of NPs having regulation on Nrf2 antioxidant pathway are overviewed. Moreover, regulatory effects of NPs on this pathway for health-promoting effects are summarized. Furthermore, structure-activity relationship of NPs for health-promoting effects by regulating the pathway is preliminarily discussed. Otherwise, the prospects on future work for regulation of NPs on this pathway are proposed. This review is beneficial to well-understanding of underlying mechanisms for health-promoting effects of NPs from the view angle of Nrf2 antioxidant pathway, and provides a theoretical basis for the development and utilization of NPs in promoting human health.

## Graphical Abstract

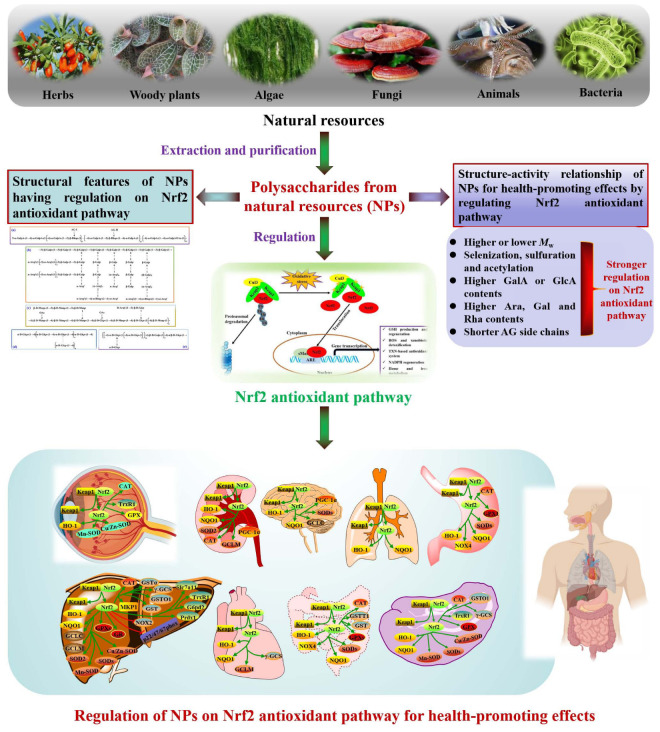

## Introduction

**FIGURE 1:**
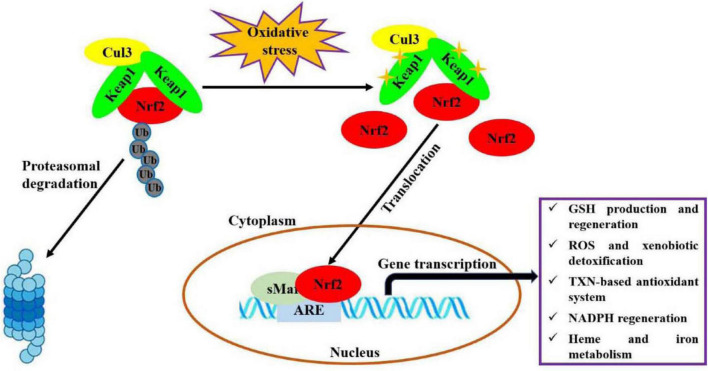
Molecular mechanism of Nrf2 signaling pathway regulating oxidative stress (7, 8). This figure is adapted from Transcriptional Regulation by Nrf2 by Claudia Tonelli et al., and NRF2, a Transcription Factor for Stress Response and Beyond by He et al., under CC BY 4.0.

Oxidative stress, an imbalance between production of oxidants and antioxidant defenses, participates in the occurrences and progressions of many diseases (1). Nuclear factor erythroid 2-related factor 2 (Nrf2) is one of the most important endogenous anti-oxidative stress pathways, which has been demonstrated to involve in modulating oxidative stress for maintaining body health, like cardioprotection (2), neuroprotection (3), anti-aging (4), gastrointestinal protection (5), and kidney protection (6). As shown in Figure 1 (7, 8), under basal conditions, Nrf2 binds to Kelch-like epichlorohydrin-associated protein-1 (Keap1) in the cytoplasm through Cul3 ubiquitin ligase containing E3 to maintain cell homeostasis. In response to oxidative stress, Nrf2 is activated upon dissociation from Keap1. Then, Nrf2 translocates quickly into nucleus and forms a necessary region for the dimer by binds to small musculoaponeurotic fibrosarcoma oncogene homolog (sMaf) protein. Subsequently, this region binds to antioxidant response elements (ARE) and activates the expressions of target genes, thereby regulates the transcriptional activities of phase II metabolic enzymes, antioxidant enzymes or drug transporters, for restoring intracellular redox homeostasis. Recently, a variety of natural products, such as polyphenols, flavonoids and polysaccharides, have been considered as modulators of Nrf2 antioxidant pathway (9, 10).

Polysaccharides, a kind of biological macromolecules, are widely distributed in natural sources such as plants, algae and animals (11). Polysaccharides have attracted increasing attention owing to their diverse health-promoting effects, non-toxicity, extensive accessibility and renewability (12). Polysaccharides from natural resources (NPs) have been reported to play key roles in regulating excessive oxidative stress (13). In the past few decades, regulations of NPs on Nrf2 antioxidant pathway have been extensively studied in their health-promoting effects, such as liver protection (14), antioxidant (15), gastrointestinal protection (16), anti-diabetic (17), anti-aging (18), cardioprotection (19), lung protection (20), kidney protection (21), neuroprotection (22), anti-inflammation (23), immunomodulation (24), anti-depression (25), anti-cancer (26), improving reproductive function (27), anti-radiation (28), and anti-atherosclerosis (29). However, the information concerning regulation of NPs on Nrf2 antioxidant pathway is scattered, and NPs show different regulatory behaviors in their different health-promoting processes. Therefore, it is necessary to draw a summary on the recent developments on health-promoting effects of NPs from the viewing angle of regulation on Nrf2 antioxidant pathway.

In this review, structural features of NPs, having regulation on Nrf2 antioxidant pathway, from herbs, woody plants, algae, fungi, animals and bacteria, are overviewed. Moreover, regulatory effects of these NPs on the pathway for health-promoting effects *in vitro* and *in vivo* are systematically summarized. Furthermore, influences of structural characteristics like molecular weight (*M*_*w*_), functional group, monosaccharide composition and side chains on the regulatory effects of NPs on Nrf2 antioxidant pathway are preliminarily discussed. Otherwise, the prospects on future work for regulation of NPs on Nrf2 antioxidant pathway are proposed.

## Structural features of NPs having regulation on Nrf2 antioxidant pathway

According to Supplementary Table 1, NPs having regulation on Nrf2 antioxidant pathway can be isolated by water extraction (16, 30–33), ultrasonic-assisted extraction (34–36), ethanol precipitation (37–41) and enzymatic hydrolysis (24, 42–44). For acquiring homogeneous fractions, NPs can be further purified by stepwise ethanol precipitation (36, 45–47) and/or column chromatography (31, 42, 43, 48–53). Structural features including *M*_*w*_, monosaccharide composition, glycosidic bond types, backbone, and side chains of the obtained NPs are shown in Supplementary Table 1.

### Structural features of NPs from herbs

In terms of NPs having regulation on Nrf2 antioxidant pathway from herbs, *M*_*w*_ values of them have been determined to range from 2.273 to 2,617 kDa (41, 54–56). The polysaccharides were composed of fucose (Fuc), ribose (Rib), mannose (Man), glucose (Glc), arabinose (Ara), galactose (Gal), rhamnose (Rha), xylose (Xyl), glucuronic acid (GlcA), galacturonic acid (GalA), glucosamine (GlcN), and/or galactosamine (GalN) (18, 56–58). Moreover, Glc, Ara, Gal, and Rha were four monosaccharide types widely discovered in the polysaccharides (18, 41, 54, 55, 57–62).

Man*p*, Glc*p*, Rha*p*, Ara*f*, Ara*p*, Gal*p*, GalA*p*, and Rib*p* sugar residues have been detected in the NPs from herbs. Man*p* residues exhibited as T-Man*p*-(1→, →3)-Man*p*-(1→, →4)-Man*p*-(1→, →6)-Man*p*-(1→, →3,6)-Man*p*-(1→ and →4,6)-Man*p*-(1→ (16, 47, 63). Glc*p* residues revealed as T-Glc*p*-(1→, →3)-Glc*p*-(1→, →4)-Glc*p*-(1→, →6)-Glc*p*-(1→, →2,4)-Glc*p*-(1→, →2,6)-Glc*p*-(1→, →3,4)-Glc*p*-(1→, →3,6)-Glc*p*-(1→ and →4,6)-Glc*p*-(1→ (16, 47, 57, 63). Rha*p* residues displayed as T-Rha*p*-(1→, →2)-Rha*p*-(1→, →3)-Rha*p*-(1→ and →2,4)-Rha*p*-(1→ (48, 54, 55, 57, 62, 64–66). Ara*f* and Ara*p* residues expressed as T-Ara*f*-(1→, →2)-Ara*f*-(1→, →4)-Ara*f*-(1→, →5)-Ara*f*-(1→, →3,5)-Ara*f*-(1→ and T-Ara*p*-(1→ (47, 48, 54, 55, 57, 62–66). Gal*p* residues showed as T-Gal*p*-(1→, →3)-Gal*p*-(1→, →4)-Gal*p*-(1→, →6)-Gal*p*-(1→, →2,6)-Gal*p*-(1→ and →3,6)-Gal*p*-(1→ (47, 48, 54, 55, 57, 62, 64–66). GalA*p* residues manifested as T-GalA*p*-(1→ and →4)-GalA*p*-(1→ (48, 54, 55, 62, 66). Rib*p* residue exhibited as →4)-Rib*p*-(1→ (47). Moreover, T-Ara*f*-(1→ (48, 54, 55, 57, 62–65), →5)-Ara*f*-(1→ (48, 54, 55, 62, 64–66), →3)-Gal*p*-(1→ (48, 55, 57, 62, 64–66), →3,6)-Gal*p*-(1→ (48, 54, 55, 62, 64–66) and →4)-GalA*p*-(1→ (48, 54, 55, 62, 66) were five frequently determined residues in the polysaccharides.

Backbone of some polysaccharides with regulation on Nrf2 antioxidant pathway from herbs were composed of T-α-D-Glc*p*-(1→ (57), →4)-α/β-D-Glc*p*-(1→ (20, 63, 67–69), →6)-α-D-Glc*p*-(1→ (57), →3,4)-α-D-Glc*p*-(1→ (57), →3)-β-D-Gal*p*-(1→ (64, 65), T-α-D-Ara*f*-(1→ (57), →4)-β-D-Man*p*-(1→ (20, 40, 63, 67, 68), →4)-α-GalA*p*-(1→ (48, 54) and/or →2,4)-α-Rha*p*-(1→ (48) units as well as homogalacturonan (HG) (55, 62, 66) and/or rhamnogalacturonan I (RG-I) (62, 66) structures. While, side chains of them were made up of →3)-β-D-Glc*p*-(1→ (20, 63, 67, 68), →3)-β-D-Man*p*-(1→ (20, 63, 67, 68), →4)-α-D-Glc*p*-(1→ (69), →5)-β-D-Ara*f*-(1→ (64, 65), →5)-α-L-Ara*f*-(1→ (62), →2)-β-D-Ara*f*-(1→ (64, 65), →6)-β-D-Gal*p*-(1→ (64, 65), →3)-β-D-Gal*p*-(1→ (64, 65), →2,4)-α-L-Rha*p*-(1→ (64, 65), T-α-L-Ara*f*-(1→ (64, 65) and/or →3)-α-Rha*p*-(1→ (48) as well as RG-I (55), type I arabinogalactan (AG-I) (62, 66) and/or type II arabinogalactan (AG-II) (48, 62, 66) structures, which branched at O-6 or C-4 position of backbones.

### Structural features of NPs from woody plants

To NPs having regulation on Nrf2 antioxidant pathway from woody plants, *M*_*w*_ of them were in the range of 4.568–3,440 kDa (70–74). The polysaccharides consisted of Fuc, Rib, Man, Glc, Ara, Gal, Rha, Xyl, GlcA, GalA, mannuronic acid (ManA), GlcN, and/or GalN (30, 75–78). Moreover, Man, Glc, Ara, Gal, and Rha were five monosaccharide types widely founded in the polysaccharides (45, 46, 76, 79–86).

Ara*f*, Ara*p*, Rha*p*, Gal*p*, Glc*p*, Man*p*, Xyl*p*, GalA*p*, and GlcA*p* sugar residues have been determined in the polysaccharides from woody plants. Ara*f* residues exhibited as T-Ara*f*-(1→, →2)-Ara*f*-(1→, →4)-Ara*f*-(1→, →5)-Ara*f*-(1→, →2,5)-Ara*f*-(1→ and →3,5)-Ara*f*-(1→ (30, 46, 73, 74). Ara*p* residues reflected as T-Ara*p*-(1→, →4)-Ara*p*-(1→, →3,4)-Ara*p*-(1→ and →2,3,4)-Ara*p*-(1→ (73, 74, 87–90). Rha*p* residues showed as T-Rha*p*-(1→, →2)-Rha*p*-(1→, →3)-Rha*p*-(1→ and →2,4)-Rha*p*-(1→ (30, 45, 87–90). Gal*p* residues exerted as T-Gal*p*-(1→, →2)-Gal*p*-(1→, →3)-Gal*p*-(1→, →4)-Gal*p*-(1→, →6)-Gal*p*-(1→, →2,6)-Gal*p*-(1→, →3,4)-Gal*p*-(1→, →3,6)-Gal*p*-(1→ and →4,6)-Gal*p*-(1→ (30, 45, 70, 87, 88, 91–96). Glc*p* residues revealed as T-Glc*p*-(1→, →2)-Glc*p*-(1→, →4)-Glc*p*-(1→, →6)-Glc*p*-(1→, →3,4)-Glc*p*-(1→ and →4,6)-Glc*p*-(1→ (49, 50, 93, 94). Man*p* residues behaved as T-Man*p*-(1→, →2)-Man*p*-(1→, →4)-Man*p*-(1→, →6)-Man*p*-(1→ and →3,6)-Man*p*-(1→ (46, 73, 74, 92). Xyl*p* residues manifested as T-Xyl*p*-(1→, →3)-Xyl*p*-(1→ and →4)-Xyl*p*-(1→ (73, 74, 87, 88). GalA*p* residues appeared as T-GalA*p*-(1→, →4)-GalA*p*-(1→, →2,4)-GalA*p*-(1→, →3,4)-GalA*p*-(1→ and →4,6)-GalA*p*-(1→ (77, 78, 91). GlcA*p* residue expressed as T-GlcA*p*-(1→ (91). Moreover, →4)-Glc*p*-(1→ (49, 50, 70, 73, 74, 87, 88, 91–96), T-Glc*p*-(1→ (49, 50, 70, 73, 74, 89–96), T-Ara*f*-(1→ (30, 45, 72–74, 91, 93–96) and →3,4)-Gal*p*-(1→ (30, 72, 77, 78, 91–96) were four residues commonly detected in the polysaccharides.

Backbone of some polysaccharides with regulation on Nrf2 antioxidant pathway from woody plants were comprised of →2)-α-D-Glc*p*-(1→ (49, 50), →4)-α-D-Glc*p*-(1→ (49, 50, 70, 95, 96), →6)-β-D-Glc*p*-(1→ (89, 90), →3)-α/β-D-Gal*p*-(1→ (72, 77, 78, 95, 96), →4)-β-D-Gal*p*-(1→ (70), →3,4)-α-D-Gal*p*-(1→ (77, 78), →3)-β-D-Ara*p*-(1→ (77, 78), →4)-α-L-Ara*p*-(1→ (89, 90), →3,4)-α-L-Ara*p*-(1→ (89, 90), →3,6)-Man*p*-(1→ (73, 74), →3)-α-L-Rha*p*-(1→ (89, 90), →2,4)-α-L-Rha*p*-(1→ (30) and/or →4)-α-D-GalA*p*-(1→ (30, 93, 94). While, the side chains of them were composed of α/β-D-Glc*p*-(1→ (49, 50, 70, 89, 90, 95, 96), →6)-α-D-Glc*p*-(1→ (49, 50), β-D-Gal*p*-(1→ (72), →6)-α-D-Gal*p*-(1→ (77, 78), →3,5,6)-β-D-Gal*f*-(1→ (72), α-D-Man*p*-(1→ (70), →6)-β-D-Man*p*-(1→ (77, 78), α-L-Ara*f*-(1→ (30, 72, 95, 96),→5)-α-L-Ara*f*-(1→ (30), →3,5)-α-L-Ara*f*-(1→ (30), and/or →4)-α-D-GalA*p*-6-OMe-(1→ (77, 78) residues, which branched at O-2, O-3, O-4, O-5, O-6, or C-4 position of backbones.

### Structural features of NPs from algae

Regarding to NPs having regulation on Nrf2 antioxidant pathway from algae, structural features of them from *Laminaria japonica* (97, 98), *Enteromorpha prolifera* (24, 31), *Sargassum fusiforme* (99), *Sargassum kjellmanianum* (17), and *Hizikia fusiforme* (44) have been characterized. Their *M*_*w*_ values ranged from 4.929 to 250 kDa (24, 97). They were made up of Fuc, Man, Rha, Ara, Gal, Glc, Xyl, GlcA, GalA, ManA, and guluronic acid (GulA) (17, 44, 99). Comparatively, Fuc and Rha were two monosaccharide types widely detected in the polysaccharides (31, 44, 97, 99). Glycosidic bond types of above-mentioned polysaccharides have yet been ascertained. ESI-CID-MS/MS and NMR analysis have indicated that the sulfated polysaccharide from *Enteromorpha prolifera* possessed a backbone consisting of D-GlcUA*p*-α-(1→4)-3-sulfate-L-Rha*p*-β-(1→4)-3-sulfate-L-Rha*p* and D-GlcUA*p*-α-(1→4)-3-sulfate-L-Rha*p*-β-(1→4)-D-Xyl*p*-β-(1→4)-3-sulfate-L-Rha*p* (100).

### Structural features of NPs from fungi

For NPs having regulation on Nrf2 antioxidant pathway from fungi, *M*_*w*_ of them were in the range of 1.206–3,011.47 kDa (39, 51, 52, 101, 102). The polysaccharides were composed of Fuc, Man, Ara, Rha, Gal, Glc, Xyl, Rib, GalA, and GlcA (39, 103, 104). Moreover, Man, Gal, and Glc were three monosaccharide types commonly determined in the polysaccharides (32–35, 103–113).

Ara*f*, Ara*p*, Rha*p*, Gal*p*, Glc*p*, Man*p*, Xyl*p*, GalA*p*, GlcA*p*, and Rib*p* sugar residues have been characterized in the polysaccharides from fungi. Man*p* residues expressed as T-Man*p*-(1→, →2)-Man*p*-(1→, →3)-Man*p*-(1→, →4)-Man*p*-(1→ and →6)-Man*p*-(1→ (51, 52, 103). Glc*p* residues showed as T-Glc*p*-(1→, →3)-Glc*p*-(1→, →4)-Glc*p*-(1→, →6)-Glc*p*-(1→, →2,4)-Glc*p*-(1→, →3,4)-Glc*p*-(1→, →3,6)-Glc*p*-(1→ and →4,6)-Glc*p*-(1→ (32, 33, 51, 52, 103). Gal*p* residues revealed as T-Gal*p*-(1→, →2)-Gal*p*-(1→, →3)-Gal*p*-(1→, →4)-Gal*p*-(1→, →6)-Gal*p*-(1→, →2,6)-Gal*p*-(1→, →3,6)-Gal*p*-(1→ and →4,6)-Gal*p*-(1→ (109, 111–113). Rha*p* residues exhibited as →4)-Rha*p*-(1→ and →6)-Rha*p*-(1→ (51, 52, 101). Ara*p*, Xyl*p* and GalA*p* residues displayed as →3)-Ara*p*-(1→ (111), T-Xyl*p*-(1→ (51, 52) and →4)-GalA*p*-(1→ (112, 113), successively. Moreover, T-Glc*p*-(1→ (32, 33, 51, 52, 103, 109, 112, 113), →3)-Glc*p*-(1→ (51, 52, 103, 110–113), →6)-Glc*p*-(1→ (101, 103, 110–113) and →6)-Gal*p*-(1→ (32, 33, 39, 101, 103, 109–111) were four residues commonly detected in the polysaccharides.

Backbone of some polysaccharides with regulation on Nrf2 antioxidant pathway from fungi were made up of →3)-Glc*p*-(1→ (51, 52, 111–113), →4)-Glc*p*-(1→ (32, 33, 39), →6)-β-D-Glc*p*-(1→ (111–113), →3,4)-Glc*p*-(1→ (51, 52), →1,4)-Glc*p*-(6→ (39), →3)-α-D-Gal*p*-(1→ (111), →4)-α-Gal*p*-(1→ (112, 113), →6)-Gal*p*-(1→ (32, 33, 39), →2)-α-Man*p*-(1→ (112, 113) and/or →4)-α-Man*p*-(1→ (112, 113). While, side chains of them were comprised of α/β-Glc*p*-(1→ (32, 33, 39, 112, 113), →3)-β-Glc*p*-(1→ (111–113), →6)-β-Glc*p*-(1→ (112, 113), T-α-D-Gal*p*-(1→ (111),→4)-α-Gal*p*-(1→ (112, 113), →3)-α-D-Man*p*-(1→ (111), →6)-β-D-Man*p*-(1→ (32, 33), →1)-Rha*f*-(2→ (39), →3)-α-L-Ara*p*-(1→ (111) and/or →4)-α-GalA*p*-(1→ (112, 113) units, which branched at O-3 and/or O-6 positions.

### Structural features of NPs from animals and bacteria

In terms of NPs having regulation on Nrf2 antioxidant pathway from animals, structural features of polysaccharides from *Holothuria leucospilota* (114), *Acaudina leucoprocta* (115), and *Ostrea talienwhanensis* Crosse (42, 43) have been determined. Polysaccharide with a *M*_*w*_ of 52.80 kDa from *Holothuria leucospilota* was composed of GalN, Fuc, GlcA, Gal, Glc, and Xyl in a mass ratio of 39.08: 35.72: 10.72: 8.43: 4.23: 1.83 (114). Polysaccharide with a *M*_*w*_ of 202 kDa from *Acaudina leucoprocta* consisted of Man, GlcN, Rha, GlcA, GalN, Gal, and Fuc in a mass ratio of 2.04: 1.30: 3.57: 5.70: 18.73: 15.12: 65.81 (115). Polysaccharide with a *M*_*w*_ of 58 kDa from *O. talienwhanensis* Crosse was solely made up of Glc, which contained T-Glc*p*-(1→, →3)-Glc*p*-(1→, →4)-Glc*p*-(1→, →6)-Glc*p*-(1→, →2,4)-Glc*p*-(1→ and →4,6)-Glc*p*-(1→ residues (42, 43).

Regarding to NPs having regulation on Nrf2 antioxidant pathway from bacteria, structural features of high (37, 38) and low (53) Fuc polysaccharides from *Bacillus megaterium* have been characterized. The former was composed of Fuc, Glc, Man, Gal and GlcNAc in a relative percentage of 41.9: 26.6: 15.8: 12.2: 3.5, which possessed a backbone consisted of →4,6)-α-D-Man*p*-(1→, →2,4)-α-D-Man*p*-(1→, →4)-β-D-Glc*p*-(1→, →2,4)-β-D-Glc*p*-(1→ and →4)-β-D-GlcNAc with a branch composed of →2,4)-β-D-Gal*p*-(1→, →4)-β-D-Gal*p*-(1→ and →3)-α-L-Fuc4SO3*p*-(1→ (37, 38). The latter was composed of Gal, Ara, Man, Glc, Fuc and GlcNAc in a relative percentage of 37.6: 20.2: 19.3: 14.0: 4.9: 4.0, which had a backbone consisted of →4,6)-α-D-Man*p*-(1→, →4)-α-D-Man*p*-(1→, →4,6)-β-D-Glc*p*-(1→ and →2,4)-β-D-Glc*p*-(1→ with a branch composed of →1)-β-D-GlcNAc*p*, →1)-α-L-Fuc4SO3*p*, →4)-β-D-Gal*p*(1→, →4,6)-β-D-Gal*p*-(1→, →2,4)-β-D-Gal*p*-(1→, →3,4)-β-L-Ara*p*-(1→ and →3)-β-L-Ara*p*-(1→ (53).

### General information on structural features of NPs having regulation on Nrf2 antioxidant pathway

With above-mentioned summarizations, it could be concluded that the *M*_*w*_ of NPs having regulation on Nrf2 antioxidant pathway were in the range of 1.206–3,440 kDa. The NPs were mostly composed of Fuc, Rha, Ara, Gal, Glc and/or Man, and frequently consisted of T-Ara*f*-(1→, →5)-Ara*f*-(1→, →3)-Gal*p*-(1→, →6)-Gal*p*-(1→, →3,4)-Gal*p*-(1→, →3,6)-Gal*p*-(1→, T-Glc*p*-(1→, →3)-Glc*p*-(1→, →4)-Glc*p*-(1→, →6)-Glc*p*-(1→ and →4)-GalA*p*-(1→ residues. Moreover, →4)-Glc*p*-(1→, →6)-Glc*p*-(1→, →3)-Gal*p*-(1→ and →4)-D-Man*p*-(1→ residues were commonly detected in their backbones, while α-L-Ara*f*-(1→, →5)-α-L-Ara*f*-(1→ and →6)-β-D-Gal*p*-(1→ residues were usually found in side chains of NPs from herbs and woody plants. Some possible repeating structural units of NPs having regulation on Nrf2 antioxidant pathway, such as pectin, arabinogalactan, 2-*O*-acetylglucomannan, glucan and glucogalactan, have been speculated. A predicted structure of the repeating units for pectin purified from *Codonopsis tangshen* roots comprised HG as the backbone and RG-I structure as the side chains (55). An arabinogalactan structure from *Lycium ruthenicum* fruits possessed a backbone of →3)-β-Gal*p*-(1→ residues, with branches of →5)-β-D-Ara*f*-(1→, →2)-β-D-Ara*f*-(1→, →6)-β-D-Gal*p*-(1→, →3)-β-D-Gal*p*-(1→, →2,4)-α-L-Rha*p*-(1→ and T-α-L-Ara*f*-(1→ at O-6 position (64). A 2-*O*-acetylglucomannan from *Dendrobium officinale* stem had a backbone of →4)-β-D-Man*p*-(1→ and →4)-β-D-Glc*p*-(1→ residues, with branches at O-6 consisting of →3)-β-D-Glc*p*-(1→ and →3)-β-D-Man*p*-(1→, and substituted with acetyl groups at O-2 (63). A glucan units from *Apios americana* tubers was characterized to possess a main chain of →4)-α-D-Glc*p*-(1→ residues with a branched →4)-α-D-Glc*p*-(1→ chain (69). A glucogalactan from *Anoectochilus zhejiangensis* was determined to have a backbone consisted of →4)-β-D-Gal*p*-(1→, →4,6)-α-D-Glc*p*-(1 → and →4)-α/β-D-Glc*p*-(1→, which branched with a single α-D-Glc*p*-(1→ at O-6 position (70).

However, the obtained purified NPs usually exhibited different structural features, owing to different methods and protocols used in above isolation and purification processes. Acidic polysaccharides (CPP-1 and CPSP-1; CTP-1 and CTSP-1) purified respectively from roots (55) and stems (66) of *Codonopsis pilosula* and *Codonopsis tangshen* had different *M*_*w*_, monosaccharide composition, glycosidic bond types, backbone and side chains. Two purified fractions (TTP-1 and TVP) acquired from tubers (71) and vines (86) of *Tetrastigma hemsleyanum* revealed differences in *M*_*w*_ and monosaccharide composition. A low-fucose-content polysaccharide (LFC) (53) and a high-fucose-content one (HFC) (37, 38) were purified from the glucose mineral salts medium (GMSM) and one in GMSM-supplemented jute culture of *Bacillus megaterium*, and they displayed different *M*_*w*_, monosaccharide composition, glycosidic bond types, backbone and side chains. Two polysaccharides (PNP80b-2 and PNP40c-1) were purified from water extracts of *Pinus koraiensis* pine nut by ethanol (80 and 40%, respectively) precipitation and same column chromatography procedures, and they were different in *M*_*w*_, monosaccharide composition and glycosidic bond types (87–90). Two purified fractions (EPP80 and EPPS-3) from *Echinacea purpurea* were obtained by ultrasonic extraction and stepwise ethanol precipitation (36), and water extraction and column chromatography (116), respectively. EPP80 and EPPS-3 exhibited different *M*_*w*_ and monosaccharide composition. Two fractions (DRP1 and DRP2) from *Dandelion* root polysaccharides were obtained by column chromatography with water and 0.1 M NaCl elution, respectively, and they showed differences in *M*_*w*_, monosaccharide composition, glycosidic bond types and backbone (57). Five purified fractions (PS-1, PS-2, PS-3, PS-4, and PS-5) were gained from *Athyrium multidentatum* subsequently eluted with 0, 0.1, 0.2, 0.3, and 0.4 M NaCl solutions, and they possessed different *M*_*w*_ and monosaccharide composition ratios (85). Two purified polysaccharides (CPP0.05 and CPP0.1) were obtained by eluting with 0.05 M and 0.1 M NaCl from *Cyclocarya paliurus*, and they behaved differences in *M*_*w*_, monosaccharide composition, glycosidic bond types, backbone and side chains (72, 95, 96).

## Regulation of NPs on Nrf2 antioxidant pathway for health-promoting effects

### Regulation of NPs from herbs

Cell experiments have demonstrated that NPs from herbs could regulate Nrf2 antioxidant pathway for liver protection (14, 117–120), kidney protection (59, 60), lung protection (20), neuroprotection (22, 65, 121–123), cardioprotection (19, 124, 125), gastrointestinal protection (48, 55, 61, 62, 66, 126–128), anti-oxidation (85, 129–134), anti-diabetic (135–137), anti-aging (138–141), anti-inflammation (67, 69), anti-radiation (28, 142), and immunomodulation (143), as illustrated in Table 1.

**TABLE 1:** Regulation of NPs from herbs on Nrf2 antioxidant pathway for health-promoting effects.

Polysaccharide source	Experimental model	Health-promoting effects	Regulation on Nrf2 antioxidant pathway	Determination method	References
*Lycium barbarum*	Hyperoxia-induced mice	Lung protection	Activities and protein expressions of Nrf2 and HO-1 in lung tissues ↑; protein expression of Keap1 in lung tissues ↓; protein expressions of Nrf2 in PMVECs isolated from lung ↑	Assay kits and WB	(158)
	Ethanol-induced L02 cells	Liver protection	Protein expression of nuclear Nrf2 ↑; protein expression of cytosol Nrf2 ↓	WB	(117)
			Protein expression of HO-1, NQO1 and GCLC along with nuclear Nrf2 ↑; protein expression of cytosol Nrf2 ↓	WB	(118)
	H_2_O_2_-induced chondrocytes	Anti-aging	mRNA and protein expressions of Nrf2, HO-1 and NQO1 ↑	WB and RT-PCR	(138)
	H_2_O_2_-induced ARPE-19 cells		Protein expressions of HO-1 and nuclear Nrf2 ↑	WB	(139)
	High-fat diet-induced mice	Anti-diabetic	Protein expressions of p-Nrf2/Nrf2, HO-1, SOD2 and CAT in liver tissues ↑	WB	(135)
	Palmitate-induced HepG2 cells		Protein expressions of p-Nrf2/Nrf2, HO-1, SOD2 and CAT ↑; nuclear translocation of p-Nrf2 ↑	WB and IF	
	Light exposure-induced mice	Anti-oxidation	mRNA expressions of Nrf2 and TrxR1 in retinas ↑	RT-PCR	(163)
	ID-8 cells bearing-mice	Anti-cancer	mRNA and protein expressions of Keap1, Nrf2 and HO-1 in liver and kidney tissues ↑	WB and RT-PCR	(168)
	UVB-induced HSF cells	Anti-radiation	Protein expressions of Nrf2 and p-Nrf2 ↑	WB	(28)
	UVB-induced HaCaT cells	Anti-radiation	Protein expression of SOD and nuclear Nrf2 ↑; mRNA expressions of AKR1C2, APOE, GCLC, GCLM, HBEGF, HO-1 and NQO1 ↑	WB and RT-qPCR	(142)
	Cerulein-induced mice	Anti-inflammation	Nuclear Nrf2 protein expression and HO-1 activity in pancreas ↑	Assay kit and WB	(23)
	Mycoplasma-infected splenic lymphocytes	Immunomodulation	mRNA and protein expressions of Nrf2, HO-1 and NQO1 ↑	WB and RT-PCR	(143)
	Ischemia-reperfusion-induced rats	Neuroprotection	Protein expressions of HO-1 and nuclear Nrf2 in retina ↑	WB and IF	(171)
	H_2_O_2_-induced PC12 cells	Neuroprotection	Protein expressions of Nrf2 and HO-1 ↑; mRNA expression of HO-1 ↑	WB, RT-qPCR and ChIP	(121)
	CoCl_2_-induced rats		mRNA expressions of Nrf2 and HO-1 in brain tissues ↑	RT-qPCR	
	LPS-induced rats	Kidney protection	mRNA and protein expressions of Nrf2, HO-1 and NQO1 in kidney tissues ↑; mRNA and protein expressions of Keap1 in kidney tissues ↓	WB and RT-qPCR	(154)
			mRNA and protein expressions of Nrf2 in kidney tissues ↑; mRNA and protein expressions of Keap1 in kidney tissues ↓; mRNA expressions of HO-1 and NQO1 in kidney tissues ↑	WB, RT-qPCR and IHC	(155)
	Lead-induced mice	Kidney protection	Protein expression of Keap1 in kidney tissues ↓; protein expressions of Nrf2, HO-1 and NQO1 in kidney tissues ↑	WB	(156)
	CTX-induced rats	Improving reproductive function	Protein expressions of Nrf2, HO-1 and NQO1 in ovarian tissues ↑	WB	(169)
	Ischemia/reperfusion-induced rats	Cardioprotection	Protein expressions of nuclear and cytosol Nrf2 in myocardial tissues ↑; protein expressions of HO-1 and NQO1 in myocardial tissues ↑	WB	(124)
	Hypoxia/reoxygenation-induced H9c2 cells		Protein expressions of nuclear and cytosol Nrf2 ↑; protein expressions of HO-1 and NQO1 ↑	WB and IF	
	Ischemia/reperfusion-induced H9c2 cells	Cardioprotection	Protein expression of nuclear Nrf2 ↑; protein expression of cytosol Nrf2 ↓	WB	(19)
*Dendrobium officinale*	DSS-induced mice	Liver protection	mRNA expressions of Nrf2, HO-1 and NQO1 in liver tissues ↑; protein expressions of Keap1, Nrf2 and HO-1 in liver tissues ↑	WB and RT-PCR	(67)
		Lung protection	Protein expression of nuclear Nrf2 in lung tissues ↑; protein expression of cytosol Nrf2 in lung tissues ↑; protein expressions of HO-1 and NQO1 in lung tissues ↑	WB	(20)
	LPS-induced BEAS-2B cells		Nuclear/cytosol Nrf2 ↑; protein expressions of HO-1 and NQO1 ↑	WB and IF	
	Acetaminophen-induced mice	Liver protection	Protein expression of nuclear Nrf2 in liver tissues ↑; Protein expression of cytosol Keap1 in liver tissues ↓; mRNA expressions of HO-1, NQO1, GCLC and GCLM in liver tissues ↑	WB and RT-PCR	(147)
	LPS-induced RAW264.7 cells	Anti-inflammation	mRNA expressions of Nrf2, HO-1 and NQO1 ↑; protein expressions of Keap1, Nrf2 and HO-1 ↑	WB and RT-PCR	(67)
	Ovariectomy or D-Gal-induced mice	Anti-aging	Protein expressions of hippocampal Nrf2 and HO-1 ↑	IF	(68)
	D-Gal-induced mice		mRNA expressions of Nrf2, HO-1 and NQO1 in liver tissues ↑	RT-qPCR	(18)
	ADM, ODM, ADM + H_2_O_2_ or ODM + H_2_O_2_-induced BMSCs cells	Anti-aging	mRNA and protein expressions of Nrf2 ↑; mRNA expressions of HO-1 and NQO1 ↑	WB and RT-qPCR	(140)
	Cisplatin-induced mice	Improving reproductive function	mRNA expressions of Nrf2, HO-1 and NQO1 in testis ↑; Protein expressions of HO-1 and NQO1 in testis ↑	WB and RT-PCR	(170)
	MNNG-induced rats	Gastrointestinal protection	Protein expressions of Nrf2, nuclear Nrf2, HO-1 and NQO1 in stomach tissues ↑; mRNA expressions of Nrf2, HO-1 and NQO1 in stomach tissues ↑	WB, RT-PCR and IHC	(161)
*Astragalus membranaceus*	Tilmicosin-induced rats	Liver protection	mRNA expressions of Nrf2 and HO-1 in liver tissues ↑	RT-qPCR	(149)
	CCl_4_-induced rats		mRNA expressions of Nrf2, SOD1 and GPX1 in liver tissues ↑	RT-qPCR	(150)
	AD model APP/PS1 mice	Anti-aging	mRNA and protein expressions of Keap1 in brain tissues ↓; mRNA expression of Nrf2 in brain tissues ↑; protein expression of nuclear Nrf2 in brain tissues ↑; protein expression of cytosol Nrf2 in brain tissues ↓	WB, RT-PCR and IF	(167)
	Oxalate-induced HK-2 cells	Kidney protection	Protein expressions of Nrf2, SOD1 and CAT ↑; protein expression of Keap1 ↓	WB	(60)
	Adjuvant arthritis rats	Cardioprotection	mRNA expressions of Keap1, MAF and Nrf2 in heart tissues ↓; protein expressions of HO-1 and γ-GCS in heart tissues ↓	RT-qPCR	(159)
	RSL3-induced Caco-2 cells	Gastrointestinal protection	Protein expressions of Nrf2 and HO-1 ↓	WB	(127)
	DSS-induced mice		Protein expressions of Nrf2 and HO-1 ↓		
*Echinacea purpurea*	Ethanol-induced mice	Liver protection	Protein expressions of Nrf2, HO-1 and NQO1 in liver tissues ↑	WB	(36)
	CCl_4_-induced mice		Protein expressions of Nrf2 and HO-1 in liver tissues ↑; protein expressions of Keap1 in liver tissues ↓	WB	(116)
Dandelion root	Acetaminophen-induced mice	Liver protection	Protein expressions of Nrf2, HO-1 and NQO1 in liver tissues ↑; protein expressions of Keap1 in liver tissues ↓	ELISA	(57)
*Sagittaria sagittifolia*	Isoniazid + rifampicin-induced mice	Liver protection	Protein and mRNA expressions of Nrf2, HO-1 and GCLC in liver tissues ↑; protein and mRNA expressions of Keap1 in liver tissues ↓	WB, RT-PCR and IHC	(151)
	Isoniazid + rifampicin-induced HepG2 cells	Liver protection	Protein and mRNA expressions of Nrf2 ↑; protein and mRNA expressions of Keap1 ↓	WB and RT-PCR	(119)
	Methionine and choline deficient diet-induced mice	Liver protection	Protein expressions of Nrf2 in liver tissues ↑	WB and IHC	(152)
	Mixture of Cd + Cr + Pb + Mn + Zn + Cu-induced mice	Liver protection	Protein expressions of Nrf2 and NQO1 in liver tissues ↑; protein expression of HO-1 in liver tissues ↓	WB, RT-qPCR and IHC	(14)
	Mixture of Cd + Cr + Pb + Mn + Zn + Cu-induced L02 cells		Protein expressions of Nrf2, HO-1 and NQO1 ↓; mRNA expressions of Nrf2 and HO-1 ↓		
*Salvia miltiorrhiza*	LPS-induced mice	Liver protection	Protein expressions of Nrf2 and HO-1 in liver tissues ↑	WB	(153)
	Florfenicol-induced chicks	Kidney protection	mRNA and protein expressions of Nrf2 and HO-1 in kidney tissues ↑; mRNA expression of NQO1 in kidney tissues ↑	WB and RT-qPCR	(21)
*Panax notoginseng*	Ethanol-induced mice	Liver protection	mRNA expressions of Nrf2, NQO1 and Cu/Zn-SOD in liver tissues ↑; mRNA and protein expressions of CAT in liver tissues ↓; protein expression of Nrf2 in liver tissues ↑	WB and RT-PCR	(54)
*Triticum aestivum* sprout	Ethanol-induced mice	Liver protection	mRNA expressions of p67phox, p47phox and p22phox in liver tissues ↓; mRNA expressions of Nrf2 and HO-1 in liver tissues ↑	RT-PCR	(148)
*Dicliptera chinensis*	High-fat diet-induced mice	Liver protection	Protein expression of Nrf2 in liver tissues ↑	WB	(41)
*Angelica sinensis*	5-Fu-induced mice	Liver protection	Protein expressions of Nrf2 and HO-1 along with nuclear Nrf2 in liver tissues ↑; protein expressions of Keap1 and cytosol Nrf2 in liver tissues ↓	WB, IHC and IF	(120)
	5-Fu-induced MIHA cells		Protein expressions of Nrf2 and HO-1 along with nuclear Nrf2 ↑; protein expressions of Keap1 and cytosol Nrf2 ↓	WB and IF	
*Athyrium multidentatum*	D-Gal-induced mice	Anti-aging	mRNA and protein expressions of Nrf2 and HO-1 in liver tissues ↑	WB and RT-PCR	(84)
	H_2_O_2_-induced HUVECs	Anti-oxidation	mRNA expressions of Nrf2 and HO-1 ↑	RT-qPCR	(85)
*Portulaca oleracea* L.	H_2_O_2_-induced MC3T3-E1 cells	Anti-aging	Protein expressions of Keap1, Nrf2, HO-1 and NQO1 ↑	WB	(141)
*Codonopsis lanceolata*	High fat/high sucrose diet-induced mice	Anti-diabetic	Protein expressions of nuclear and cytosol Nrf2 in liver tissues ↑; protein expressions of nuclear and cytosol Keap1 in liver tissues ↓; mRNA expressions of Nrf2, HO-1 and NQO1 in liver tissues ↑	WB and RT-PCR	(58)
Pumpkin	High-fat diet + STZ-induced mice	Anti-diabetic	Protein expressions of HO-1 and nuclear Nrf2 in liver tissues ↑	WB	(56)
*Abelmoschus esculentus*	High-fat diet + STZ-induced mice	Anti-diabetic	Protein expressions of Nrf2, HO-1 and SOD2 kidney tissues ↑	WB	(165)
			Protein expressions of HO-1, SOD2 and Nrf2 liver tissues ↑; protein expressions of NOX2 in liver tissues ↓	WB and IHC	(166)
*Cassia* seeds	High glucose-induced HRECs	Anti-diabetic	Protein expressions of Nrf2 and HO-1 ↑; mRNA expression of HO-1 ↑	WB and RT-qPCR	(146)
*Polygonatum sibiricum*	High glucose-induced ARPE-19 cells	Anti-diabetic	Protein expressions of HO-1 and nuclear Nrf2 ↑	WB	(136)
	High-glucose- and high-insulin-induced 3T3-L1 adipocytes	Anti-diabetic	Protein expressions of Nrf2 and HO-1 ↑		(137)
	MPTP-induced mice	Neuroprotection	Protein expressions of Nrf2 and NQO1 ↑	WB	(22)
	MPP+-induced N2a cells		Protein expressions of Nrf2, HO-1, NQO1, GCLC and GCLM ↑		
*Codonopsis pilosula*	Ethanol-induced mice	Anti-oxidation	mRNA expressions of Keap1 and Nrf2 in liver tissues ↑	RT-PCR	(164)
	H_2_O_2_-induced RAW264.7 cells	Anti-oxidation	Protein expressions of Keap1 ↓; protein expressions of Nrf2, HO-1, NQO1, GCLM and GCLC ↑	WB	(131)
	H_2_O_2_-induced IPEC-J2 cells	Gastrointestinal protection	mRNA expressions of GPX, SOD1, CAT, Nrf2, NQO1 and HO-1 ↑	RT-qPCR	(55)
			mRNA expressions of GPXs, SOD1 and CAT ↑		(66)
*Taraxacum mongolicum*	Jian carp	Anti-oxidation	mRNA expression of Keap1 in spleen ↓; mRNA expressions of Nrf2, HO-1, Cu/Zn-SOD, GPX, CAT and Mn-SOD in spleen ↑	RT-qPCR	(15)
*Taraxacum officinale*	LPS-induced RAW264.7 cells	Anti-oxidation	Protein expressions of Nrf2 and HO-1 ↑	WB	(132)
*Alfalfa*	H_2_O_2_-induced MEFs cells	Anti-oxidation	Protein expressions of nuclear and cytosol Nrf2 ↑	WB and IF	(133)
*Hosta ventricosa*	Tert-butyl hydroperoxide-induced HepG2 cells	Anti-oxidation	mRNA expressions of Keap1, Nrf2, HO-1, NQO1 and GST ↑; protein expressions of HO-1, NQO1 and nuclear Nrf2 ↑; protein expression of cytosol Nrf2 ↓	WB and RT-qPCR	(130)
*Cistanche deserticola*	H_2_O_2_-induced HEMs	Anti-oxidation	Protein expressions of nuclear and cytosol Nrf2 along with nuclear/cytosol Nrf2 ↑; protein expression of HO-1 ↑	WB and IF	(134)
Fermented wheat bran	Zebrafish	Anti-oxidation	mRNA expressions of CAT, GPX-3, GST, Nrf2 and p38 in intestines ↑	RT-qPCR	(162)
*Thymus quinquecostatus*	AAPH-induced zebrafish	Anti-oxidation	mRNA expression of Keap1 in larvae ↓; mRNA expressions of Nrf2, SOD, CAT and HO-1 in larvae ↑	RT-qPCR	(47)
Wheat germ	Oleic acid-induced HepG2 cells	Anti-oxidation	Protein expression of Nrf2, HO-1 and nuclear Nrf2 ↑	ELISA and WB	(129)
*Apios americana*	LPS-induced RAW264.7 cells	Anti-inflammation	Protein expressions of Keap1 and Nrf2 ↑	WB	(69)
*Polygonatum cyrtonema*	LPS and CUMS-induced mice	Anti-depression	Protein expressions of Nrf2 and HO-1 in hippocampal tissues ↑	WB and IF	(25)
*Aloe vera*	UVB-induced PC12 cells	Neuroprotection	mRNA and protein expressions of Keap1, Nrf2, GCLC and GSTP1 ↑	WB and RT-PCR	(122)
	DSS-induced mice	Gastrointestinal protection	Protein expressions of Nrf2, HO-1 and NQO1 in colon tissues ↑	WB	(40)
*Lycium ruthenicum*	OGD/R-induced primary cortical neurons	Neuroprotection	Protein expressions of HO-1 and nuclear Nrf2 ↑	WB	(65)
*Perilla frutescens*	H_2_O_2_-induced HT22 cells	Neuroprotection	Protein expressions of HO-1, NQO1 and nuclear Nrf2 ↑; protein expression of cytosol Nrf2 ↓	WB	(123)
*Potentilla anserina*	Cadmium-induced HEK293 cells	Kidney protection	Protein expressions of Nrf2 and PGC-1α↓	WB	(59)
	Cadmium-induced mice		Protein expressions of Nrf2 and PGC-1α in kidney tissues ↓		
*Momordica charantia*	STZ-induced rats	Kidney protection	Protein expressions of Nrf2 and HO-1 in kidney tissues ↑	WB	(157)
Blood cora	H_2_O_2_-induced H9c2 cells	Cardioprotection	mRNA expressions of Nrf2, HO-1, NQO1 and nuclear Nrf2 ↓; protein expressions of Nrf2 and HO-1 ↓	WB and RT-PCR	(125)
*Dendrobium fimbriatum*	DSS-induced mice	Gastrointestinal protection	Protein expression of Nrf2 in colon tissues ↑; protein expression of Keap1 in colon tissues ↓	WB	(16)
*Nelumbo nucifera* leaves	Aged mice	Gastrointestinal protection	mRNA expressions of Nrf2, SOD1, SOD2, CAT and GPX1 in jejunum and colon ↑	RT-qPCR	(48)
	H_2_O_2_-induced IPEC-J2 cells		mRNA expression of Nrf2 ↑		
*Rheum tanguticum*	Radiation-induced rats	Gastrointestinal protection	Protein expressions of nuclear and cytosol Nrf2 along with cytosol HO-1 in jejunum ↑; mRNA expressions of Nrf2, nuclear Nrf2, cytosol Nrf2, HO-1 and cytosol HO-1 in jejunum ↑	WB, RT-PCR and IHC	(128)
	Radiation-induced IEC-6 cells		Protein expressions of nuclear and cytosol Nrf2 along with cytosol HO-1 ↑	WB and IF	
*Platycodon grandiflorus*	H_2_O_2_-induced IPEC-J2 cells	Gastrointestinal protection	mRNA expressions of Nrf2, NQO1, CAT and GPX ↑	RT-qPCR	(62)
*Codonopsis tangshen*	H_2_O_2_-induced IPEC-J2 cells	Gastrointestinal protection	mRNA expressions of GPXs, SOD1, CAT, Nrf2, NQO1 and HO-1 ↑	RT-qPCR	(55)
Hemp seed	CTX-induced mice	Gastrointestinal protection	mRNA expressions of Nrf2, HO-1, NQO1, SOD and GPX in ileum tissues ↑; protein expression of Nrf2 in ileum tissues ↑; protein expression of Keap1 in ileum tissues ↓	WB and RT-qPCR	(160)
	H_2_O_2_-induced IPEC-1 cells	Gastrointestinal protection	mRNA expressions of SOD, GPX, CAT, HO-1, NQO1 and Nrf2 ↑; protein expression of Nrf2 ↑; protein expression of Keap1 ↓	WB and RT-PCR	(61)
Corn silk	H_2_O_2_-induced IEC-6 cells	Gastrointestinal protection	protein expression of Keap1 ↓; protein expressions of Nrf2 and HO-1 ↑	WB	(126)

Natural polysaccharides from herbs exerted liver protection against ethanol- (117, 118) or mixture of Cd + Cr + Pb + Mn + Zn + Cu-induced (14) L02 cells, isoniazid + rifampicin-induced HepG2 cells (119) and 5-fluorocrail (5-Fu)-induced MIHA cells (120), partly through modulating protein and mRNA expressions of Nrf2, HO-1, and NQO1, increasing protein expressions of GCLC and nuclear Nrf2, and decreasing protein and/or mRNA expressions of Keap1 and cytosol Nrf2. Those from *Astragalus membranaceus* (60) and *Potentilla anserine* (59) exhibited kidney protection on oxalate-induced HK-2 cells and cadmium-induced HEK293 cells, respectively, whose actions were related to regulation of Nrf2 protein expression, reduction of Keap1 and PGC-1α protein expressions and increment of SOD1 and CAT protein expressions. Polysaccharide from *Dendrobium officinale* showed lung protection in LPS-induced BEAS-2B cells involved with increases of HO-1 and NQO1 protein expressions as well as nuclear/cytosol Nrf2 ratio (20). NPs from herbs displayed neuroprotection against MPP+-induced N2a cells (22), H_2_O_2_- (121), UVB- (122), and OGD/R-induced (144) PC12 cells, OGD/R-induced primary cortical neurons (65), and H_2_O_2_-induced microglia BV2 cells (145) or HT22 cells (123), which were correlated with promotions of mRNA and protein expressions of Keap1, Nrf2, HO-1, NQO1, GCLC, GCLM, and GSTP1 along with nuclear Nrf2, and reduction of cytosol Nrf2 protein expression. Moreover, *Salvia miltiorrhiza* polysaccharides protected PC12 cells from OGD/R-induced ferroptosis and lipid peroxidation by activating Nrf2/HO-1 pathway (144). *Polygonatum cyrtonema* Hua polysaccharides alleviated ferroptosis in H_2_O_2_-induced microglia BV2 cells by activating Nrf2/HO-1 signaling pathway (145). NPs from herbs revealed cardioprotection on hypoxia/reoxygenation- (124), ischemia/reperfusion- (19) or H_2_O_2_-induced (125) H9c2 cells by modulating protein and mRNA expressions of Nrf2, HO-1, and NQO1 as well as nuclear and cytosol Nrf2. Those of herbs appeared gastrointestinal protection against RSL3-induced Caco-2 cells (127), H_2_O_2_- (126) or radiation-induced (128) IEC-6 cells and H_2_O_2_-induced IPEC-J2 cells (48, 55, 62, 66) or IPEC-1 cells (61), partly through modulating protein and mRNA expressions of Nrf2 and HO-1, elevating protein and mRNA expressions of NQO1, SOD, SOD1, CAT, GPX, nuclear, and cytosol Nrf2 along with cytosol HO-1, and decreasing Keap1 protein expression. Moreover, *Astragalus* polysaccharide exhibited inhibitory effect on ferroptosis in RSL3-induced Caco-2 cells and this effect was associated with the Nrf2/HO-1 pathway (127). NPs from herbs possessed anti-oxidation on H_2_O_2_-induced HUVECs (85), H_2_O_2_- (131) or LPS-induced (132) RAW264.7 cells, H_2_O_2_-induced MEFs cells (133), tert-butyl hydroperoxide- (130) or oleic acid-induced (129) HepG2 cells and H_2_O_2_-induced HEMs (134) via enhancing protein and mRNA expressions of Nrf2, HO-1, NQO1, GCLM, GCLC, and GST along with nuclear/cytosol Nrf2, and regulating protein and mRNA expressions of Keap1. Those from herbs exhibited anti-diabetic effect on palmitate-induced HepG2 cells (135), high glucose-induced ARPE-19 cells (136) or HRECs (146), and high-glucose- and high-insulin-induced 3T3-L1 adipocytes (137) by augmenting protein or mRNA expressions of p-Nrf2/Nrf2, Nrf2, HO-1, SOD2, CAT, and nuclear Nrf2 as well as nuclear translocation of p-Nrf2. NPs from herbs showed anti-aging activity against H_2_O_2_-induced chondrocytes (138), H_2_O_2_-induced ARPE-19 cells (139), ADM, ODM, ADM + H_2_O_2_, or ODM + H_2_O_2_-induced BMSCs cells (140), H_2_O_2_-induced MC3T3-E1 cells (141) through rising mRNA and/or protein expressions of Keap1, Nrf2, HO-1, and NQO1 as well as nuclear Nrf2. Polysaccharides from *Dendrobium officinale* (67) and *Apios americana* (69) produced anti-inflammation on LPS-induced RAW264.7 cells partly by adding protein and/or mRNA expressions of Keap1, Nrf2, HO-1 and NQO1. Polysaccharides from *Lycium barbarum* produced anti-radiation action on UVB-induced HSF and HaCaT cells via enlarging protein and/or mRNA expressions of Nrf2, p-Nrf2, HO-1, NQO1, GCLC, GCLM, SOD, AKR1C2, APOE, and HBEGF along with nuclear Nrf2 (28, 142). Meanwhile, *Lycium barbarum* polysaccharide caused immunomodulation in mycoplasma-infected splenic lymphocytes through increments of mRNA and protein expressions of Nrf2, HO-1, and NQO1 (143).

Animals experiments have demonstrated that NPs from herbs could regulate Nrf2 antioxidant pathway for liver protection (14, 36, 41, 54, 57, 67, 116, 120, 147–153), kidney protection (21, 59, 154–157), lung protection (20, 158), neuroprotection (22, 121), cardioprotection (124, 159), gastrointestinal protection (16, 40, 48, 127, 128, 160, 161), anti-oxidation (15, 47, 162–164), anti-diabetic (56, 58, 135, 146, 165, 166), anti-aging (18, 68, 84, 167), anti-inflammation (23), anti-depression (25), anti-cancer (168), and improving reproductive function (169, 170), as implied in Table 1.

Natural polysaccharides from herbs exerted liver protection against DSS- (67), acetaminophen- (57, 147), tilmicosin- (149), CCl_4_- (116, 150), ethanol- (36, 54, 148), isoniazid + rifampicin- (151), methionine and choline deficient diet- (152), mixture of Cd + Cr + Pb + Mn + Zn + Cu- (14), LPS- (153), high-fat diet- (41), and 5-Fu-induced (120) mice or rats, through increasing mRNA and protein expressions of Nrf2, nuclear Nrf2, NQO1, GCLC, GCLM, Cu/Zn-SOD, SOD1, and GPX1 in liver tissues, modulating protein and/or mRNA expressions of Keap1 and HO-1, and decreasing protein and/or mRNA expressions of cytosol Keap1, CAT, cytosol Nrf2, p67phox, p47phox, and p22phox in liver tissues. NPs from herbs exhibited kidney protection on LPS- (154, 155), lead- (156), florfenicol- (21), cadmium- (59), and STZ-induced (157) mice, rats or chicks via elevating mRNA and protein expressions of HO-1 and NQO1, regulating Nrf2 expression, and down-regulating mRNA and protein expressions of Keap1 and PGC-1α in kidney tissues. Those from herbs showed lung protection hyperoxia- (158) and DSS-induced (20) mice by enhancing activities and/or protein expressions of Nrf2, cytosol Nrf2, nuclear Nrf2, HO-1, and NQO1 in lung tissues as well as protein expressions of Nrf2 in PMVECs isolated from lung, and reducing protein expression of Keap1 in lung tissues. NPs from herbs reflected neuroprotection against ischemia-reperfusion- (171), CoCl_2_- (121), and MPTP-induced (22) mice or rats, which is related to increments of protein and/or mRNA expressions of nuclear Nrf2, Nrf2, HO-1 and NQO1 in retina or brain tissues. Those from herbs displayed cardioprotection on ischemia/reperfusion-induced (124) and adjuvant arthritis rats (159), involving with aggrandizement of protein expressions of nuclear and cytosol Nrf2, HO-1 and NQO1 in myocardial tissues, and declination of mRNA and/or protein expressions of Keap1, MAF, Nrf2, HO-1, and γ-GCS in heart tissues. NPs from herbs appeared gastrointestinal protection against MNNG- (161), DSS- (16, 40, 127), radiation- (128), and CTX-induced (160) mice or rats as well as aged mice (48), via up-regulating protein and/or mRNA expressions of nuclear and cytosol Nrf2, cytosol HO-1, NQO1, SOD, SOD1, SOD2, CAT, GPX, and GPX1, modulating protein and/or mRNA expressions of Nrf2 and HO-1, and down-regulating Keap1 protein expression in stomach, colon or jejunum tissues. Meanwhile, *Astragalus* polysaccharide inhibited ferroptosis of colonic tissue through Nrf2/HO-1 pathway in DSS-induced mice (127). NPs from herbs generated anti-oxidation effects on light exposure-induced mice (163), ethanol-induced mice (164), AAPH-induced zebrafish (47) as well as Jian carp (15) and zebrafish (162) through adding mRNA expressions of Nrf2, HO-1, Cu/Zn-SOD, GPX, GPX-3, CAT, SOD, Mn-SOD, GST, TrxR1, and p38, and modulating Keap1 mRNA expression in retinas, spleen or liver tissues. NPs from herbs produced anti-diabetic activity against high-fat diet- (135), high fat/high sucrose diet- (58) and high-fat diet + STZ-induced (56, 165, 166) mice via increasing protein and/or mRNA expressions of p-Nrf2/Nrf2, nuclear and cytosol Nrf2, Nrf2, HO-1, NQO1, SOD2, and CAT in liver or kidney tissues, and decreasing protein expressions of nuclear and cytosol Keap1 and NOX2 in liver tissues. Those from herbs caused anti-aging effects on ovariectomy or D-Gal-induced mice (18, 68, 84) and AD model APP/PS1 mice (167), through elevating mRNA and protein expressions of nuclear Nrf2, Nrf2, HO-1 and NQO1 in hippocampal, brain, and liver tissues, and reducing mRNA and/or protein expressions of Keap1 and cytosol Nrf2 in brain tissues. *Lycium barbarum* polysaccharide revealed anti-inflammation against cerulein-induced mice by adding nuclear Nrf2 protein expression and HO-1 activity in pancreas (23). Meanwhile, this polysaccharide implied anti-cancer action against ID-8 cells bearing-mice through up-regulation of mRNA and protein expressions of Keap1, Nrf2 and HO-1 in liver and kidney tissues (168). *Polygonatum cyrtonema* polysaccharide had anti-depression activity on LPS and CUMS-induced mice via increasing protein expressions of Nrf2 and HO-1 in hippocampal tissues (25). NPs from herbs possessed improving reproductive function against CTX-induced rats (169) and cisplatin-induced mice (170) by elevating protein and/or mRNA expressions of Nrf2, HO-1 and NQO1 in ovarian or testis tissues.

### Regulation of NPs from woody plants

Cell experiments have indicated that NPs from woody plants could regulate Nrf2 antioxidant pathway for liver protection (30, 45, 46, 50, 70, 92, 172), kidney protection (173), gastrointestinal protection (86), neuroprotection (75), cardioprotection (78), anti-aging (74), anti-diabetic (174), anti-oxidation (72, 96, 175), and anti-inflammation (71), as showed in Table 2.

**TABLE 2:** Regulation of NPs from woody plants on Nrf2 antioxidant pathway for health-promoting effects.

Polysaccharide source	Experimental model	Health-promoting effects	Regulation on Nrf2 antioxidant pathway	Determination method	References
Chestnut shell	H_2_O_2_-induced primary hepatocytes from hybrid grouper	Liver protection	mRNA expressions of GPX, Mn-SOD and Nrf2 ↑; mRNA expression of GR ↓	RT-PCR	(30)
	H_2_O_2_-induced hybrid grouper		mRNA expressions of CAT, GPX and GR in liver tissues ↑		
*Smilax china* L.	Acetaminophen-induced mice	Liver protection	Protein expressions of Nrf2, HO-1, NQO1 and GCLC along with nuclear translocation of Nrf2 in liver tissues ↑	WB and EMSA	(50)
	H_2_O_2_-induced AML12 cells		Protein expression of Nrf2 and nuclear translocation of Nrf2 ↑; mRNA and protein expressions of HO-1, NQO1 and GCLC ↑	WB and RT-PCR	
*Anoectochilus zhejiangensis*	CCl_4_-induced HepG2 cells	Liver protection	Protein expressions of Nrf2, HO-1 and NQO1 ↑	WB	(70)
*Malpighia emarginata*	High-fat diet-induced mice	Liver protection	Protein expressions of Nrf2, HO-1 and NQO1 in liver tissues ↑	WB	(79)
Wild jujube	CCl_4_-induced mice	Liver protection	Protein expressions of HO-1, GSTα and NQO1 along with nuclear Nrf2 in liver tissues ↑	WB	(80)
*Anoectochilus roxburghii*	High-fat diet-induced mice	Liver protection	Protein expressions of Nrf2, HO-1 and NQO1 in liver tissues ↑	WB	(176)
Pine nut	CCl_4_-induced mice	Liver protection	mRNA expression of Nrf2 in liver tissues ↑; protein and mRNA expression of Keap1 in liver tissues ↓; protein and mRNA expressions of HO-1, NQO1 and GCLC in liver tissues ↑; protein expressions of MKP1 and nuclear Nrf2 in liver tissues ↑; protein expression of cytosol Nrf2 in liver tissues ↓	WB and RT-PCR	(90)
			mRNA expressions of Nrf2 and HO-1 in liver tissues ↑	RT-PCR	(87)
	Ethanol-induced mice				
	Acetaminophen-induced mice				
	Ethanol-induced mice		Protein expressions of Nrf2 and HO-1 in liver tissues ↑	WB	(88)
*Sonneratia apetala*	Acetaminophen-induced mice	Liver protection	Protein expression of nuclear Nrf2 in liver tissues ↑; Protein expressions of cytosol Keap1 and Nrf2 in liver tissues ↓; protein and mRNA expressions of HO-1, NQO1, GCLC and GCLM in liver tissues ↑	WB and RT-PCR	(91)
*Schisandra chinensis*	Acetaminophen-induced mice	Liver protection	Protein expressions of Nrf2 and HO-1 in liver tissues ↑	WB	(82)
	Cyclosporin A-induced LX-2 cells		Protein expression of nuclear Nrf2 ↑		(92)
	Concanavalin A-induced mice		Protein expressions of Nrf2 and HO-1 in liver tissues ↑; protein expression of Keap1 in liver tissues ↓		(83)
	293T cells	Kidney protection	Protein expressions of Nrf2, NQO1 and HO-1, and NQO1-antioxidant response element-luciferase activity ↑; protein expressions of cytosol Keap1 and Nrf2 ↓; protein expression of nuclear Nrf2 ↑	WB and IF	(173)
*Morinda citrifolia* L.	High-fat diet-induced mice	Liver protection	Nrf2 level in liver tissues ↑	ELISA	(94)
Pomelo fruitlet	Hepatocytes isolated from High-fat diet-induced hybrid grouper	Liver protection	mRNA expressions of Nrf2, Mn-SOD, CAT and GPX ↑	RT-PCR	(172)
Mulberry fruit	Palmitic acid-induced HepG2 cells	Liver protection	mRNA expressions of HO-1, NQO1 and γ-GCL ↑; protein expressions of p-Nrf2 and nuclear Nrf2 ↑	WB and RT-PCR	(45)
Black mulberry	Palmitate-induced HepG2 cells	Liver protection	mRNA expressions of HO-1, NQO1, γ-GCL, GPX and CAT ↑; protein expressions of NQO1, p-Nrf2 and nuclear Nrf2 ↑	WB and RT-PCR	(46)
*Aronia melanocarpa*	D-Gal-induced mice	Anti-aging	Protein expressions of nuclear Nrf2 and HO-1 in brain tissues ↑	WB	(76)
*Taxus chinensis var. mairei*	D-Gal-induced mice	Anti-aging	Protein expressions of Nrf2 and SOD in brain tissues ↑	WB	(74)
	D-Gal-induced BV2 cells		Protein expressions of Nrf2 and SOD ↑		
*Opuntia milpa alta*	Alloxan-induced INS-1 cells	Anti-diabetic	Protein expressions of Nrf2 and γ-GCSc ↑	WB	(174)
*Cyclocarya paliurus*	H_2_O_2_-induced DCs	Anti-oxidation	mRNA expressions of CAT, GPX1, SOD, HO-1 and NQO1 ↑; protein expression of Nrf2 ↑; protein expression of Keap1 ↓	WB and RT-qPCR	(72)
			Protein expression of Nrf2 ↑; protein expression of Keap1 ↓	WB	(96)
*Artemisia ordosica*	LPS-induced broilers	Anti-oxidation	mRNA and protein expressions of Nrf2, GPX, CAT and SOD in liver tissues ↑; mRNA and protein expressions of Keap1 in liver tissues ↓	WB and RT-PCR	(178)
Pistachio hull	LPS-induced Nile tilapia	Anti-oxidation	mRNA expressions of Nrf2, SOD and CAT in liver tissues ↑	RT-PCR	(179)
*Chimonanthus nitens Oliv*	CTX-induced mice	Anti-oxidation	mRNA expressions of Nrf2, SOD1, CAT, GPX, NQO1 and HO-1 in liver tissues ↑; mRNA and protein expressions of Keap1 in liver tissues ↓; protein expressions of Nrf2, NQO1 and HO-1 in liver tissues ↑	WB and RT-qPCR	(177)
Rice bran	293T cells	Anti-oxidation	Protein expressions of Nrf2, NQO1 and HO-1 ↑	WB	(175)
*Tetrastigma hemsleyanum*	LPS-induced RAW264.7 cells	Anti-inflammation	Protein expressions of Keap1 and Nrf2 ↑	WB	(71)
	Ethyl carbamate-induced Caco-2 cells	Gastrointestinal protection	Protein expressions of Keap1 and Nrf2 ↑	WB	(86)
*Pyracantha fortuneana*	Mice	Immunomodulation	mRNA and protein expressions of Nrf2 in splenocytes ↑	WB and RT-PCR	(180)
Selenium-enriched green tea	Mice	Immunomodulation	mRNA and protein expressions of Nrf2 in splenocytes ↑	WB and RT-PCR	(181)
*Annona muricata*	H_2_O_2_-induced HT22 cells	Neuroprotection	Protein expressions of HO-1, NQO1 and nuclear Nrf2 ↑; protein expression of cytosol Nrf2 ↓	WB	(75)
*Fructus Aurantii*	Isoproterenol-induced rats	Cardioprotection	Protein expressions of HO-1, NQO1, GCLM and γ-GCS in cardiac muscle tissues ↑; Protein expressions of nuclear and cytosol Nrf2 in cardiac muscle tissues ↑	WB	(77)
	Hypoxia/reoxygenation-induced H9c2 cells		Protein expressions of HO-1 and Nrf2 ↓		(78)

Natural polysaccharides from woody plants exhibited liver protection against H_2_O_2_-induced primary hepatocytes from hybrid grouper (30), H_2_O_2_-induced AML12 cells (50), CCl_4_- (70) and palmitic acid-induced (45, 46) HepG2 cells, cyclosporin A-induced LX-2 cells (92), and hepatocytes isolated from high-fat diet-induced hybrid grouper (172), involving with increments of mRNA and protein expressions of p-Nrf2, nuclear Nrf2, Nrf2, HO-1, NQO1, γ-GCL, GCLC, Mn-SOD, GPX, and CAT as well as nuclear translocation of Nrf2, and reduction of GR mRNA expression. *Schisandra chinensis* polysaccharide generated kidney protection on 293T cells through increasing protein expressions of nuclear Nrf2, Nrf2, NQO1, and HO-1 along with NQO1-antioxidant response element-luciferase activity, and decreasing protein expressions of cytosol Keap1 and Nrf2 (173). NPs from *Tetrastigma hemsleyanum* showed gastrointestinal protection against ethyl carbamate-induced Caco-2 cells, by elevating protein expressions of Keap1 and Nrf2 (86). Polysaccharide from *Annona muricata* (75) caused neuroprotection on H_2_O_2_-induced HT22 cells via adding protein expressions of HO-1, NQO1 and nuclear Nrf2, and reducing cytosol Nrf2 protein expression. *Fructus Aurantii* polysaccharide produced cardioprotection against hypoxia/reoxygenation-induced H9c2 cells through lowering protein expressions of HO-1 and Nrf2 (78). *Taxus chinensis var. mairei* polysaccharide exerted anti-aging action on D-Gal-induced BV2 cells by promoting protein expressions of Nrf2 and SOD (74). Polysaccharide *Opuntia milpa alta* (174) revealed anti-diabetic activities against alloxan-induced INS-1 cells, which was related to enhancements of protein expressions of Nrf2 and γ-GCSc. NPs from woody plants displayed anti-oxidation effects on H_2_O_2_-induced DCs (72, 96) and 293T cells (175), partly by rising protein and/or mRNA expressions of Nrf2, CAT, GPX1, SOD, HO-1, and NQO1, and reducing Keap1 protein expression. *Tetrastigma hemsleyanum* polysaccharide reflected anti-inflammation on LPS-induced RAW264.7 cell via through improving protein expressions of Keap1 and Nrf2 (71).

Animal experiments have indicated that NPs from woody plants could regulate Nrf2 antioxidant pathway for liver protection (30, 50, 79, 80, 82, 83, 87, 88, 90, 91, 94, 172, 176), cardioprotection (77), anti-aging (74, 76), anti-oxidation (177–179), and immunomodulation (180, 181), as reflected in Table 2.

NPs from woody plants had liver protection against H_2_O_2_-induced hybrid grouper (30), acetaminophen-induced mice (50, 82, 87), high-fat diet-induced mice (79, 94, 176), CCl_4_-induced mice (80, 90), ethanol-induced mice (87, 88), and concanavalin A-induced mice (83), involving with increment of protein and/or mRNA expressions of nuclear Nrf2, Nrf2, HO-1, NQO1, GCLC, CAT, GSTα, GPX, GR, and MKP1 along with nuclear translocation of Nrf2, and reduction of protein and/or mRNA expressions of Keap1 and cytosol Nrf2 in liver tissues. *Fructus Aurantii* polysaccharide exerted cardioprotection against isoproterenol-induced rats via enhancing protein expressions of HO-1, NQO1, GCLM, γ-GCS, nuclear Nrf2, and cytosol Nrf2 in cardiac muscle tissues (77). Polysaccharides from *Aronia melanocarpa* (76) and *Taxus chinensis var. mairei* (74) revealed anti-aging activity on D-Gal-induced mice by up-regulating protein expressions of nuclear Nrf2, Nrf2, HO-1, and SOD in brain tissues. NPs from woody plants generated anti-oxidation effect against LPS-induced broilers (178) or Nile tilapia (179) and CTX-induced mice (177), which was related to enhancement of mRNA and protein expressions of Nrf2, NQO1, HO-1, GPX, CAT, SOD1, and SOD in liver tissues, intestines or larvae, and reduction of mRNA and protein expressions of Keap1 in liver tissues or larvae. Polysaccharides from *Pyracantha fortuneana* (180) and selenium-enriched green tea (181) reflected immunomodulation on mice through adding mRNA and protein expressions of Nrf2 in splenocytes.

### Regulation of NPs from algae

The regulations of NPs on Nrf2 antioxidant pathway from algae in cell and animal experiments are revealed in Table 3.

**TABLE 3:** Regulation of NPs from algae on Nrf2 antioxidant pathway for health-promoting effects.

Polysaccharide source	Experimental model	Health-promoting effects	Regulation on Nrf2 antioxidant pathway	Determination method	References
*Ecklonia cave*	LPS-induced mice	Lung protection	Protein expressions of Nrf2 and HO-1 in lung tissues ↑	WB	(186)
*Laminaria digitata*	H_2_O_2_-induced MRC-5 cells	Lung protection	mRNA expressions of Nrf2, HO-1, NQO1 and GCLC ↑; mRNA expression of Keap1 ↓; protein expression of nuclear Nrf2 ↑; Nuclear translocation of Nrf2 ↑	WB, RT-qPCR and IF	(183)
*Laminaria japonica*	CTX-induced mice	Liver protection	Protein expressions of Nrf2, HO-1, GCLM and NQO1 in liver or kidney tissues ↑	WB	(97)
		Kidney protection			
	Rotenone-induced rats	Anti-aging	Protein expressions of Nrf2 and PGC-1α in ventral midbrain ↑	WB	(98)
*Enteromorpha prolifera*	CCl_4_-induced mice	Liver protection	Protein expressions of p-Nrf2 and HO-1 along with p-Nrf2/Nrf2 in liver tissues ↑; mRNA expression of NQO1 in liver tissues ↑	WB and RT-qPCR	(31)
	Heat stress-induced *Gallus gallus domesticus*	Anti-oxidation	mRNA expressions of SOD2, GSTO1 and HO-1 in spleen ↑; protein expression of total Nrf2 in spleen ↑	WB and RT-qPCR	(190)
	Aflatoxin B1-induced broilers	Immunomodulation	mRNA expressions of SOD1, SOD2, GPX1, GPX3, CAT1, GSTT1, GSTO1, GSTA3, Nrf2 and HO-1 in bursa of fabricius ↑; protein expressions of Nrf2 and HO-1 in bursa of fabricius ↑	WB and RT-qPCR	(24)
	Heat stress-induced broilers	Gastrointestinal protection	mRNA expressions of Nrf2, HO-1, GPX1 and GSTT1 in duodenum ↑	RT-qPCR	(187)
*Sargassum fusiforme*	High-fat diet-induced mice	Liver protection	Protein expressions of nuclear and cytosol Nrf2 in liver tissues ↑; protein expression of Keap1 in liver tissues ↓; mRNA expressions of Nrf2, NQO1, HO-1, CAT, SOD2, Slc7a11, G6pd2, Prdx1, GPX2 and GPX4 in liver tissues ↑	WB and RT-qPCR	(185)
	Heat stress-induced *Drosophila melanogaster*	Anti-aging	mRNA expressions of CncC, HO and GCLC ↑; mRNA expression of Keap1 ↓	RT-qPCR	(99)
	D-Gal-induced mice		Protein expressions of Nrf2 and NQO1 in liver tissues ↑; mRNA and protein expressions of Keap1 in liver tissues ↑; mRNA expressions of Cu/Zn-SOD and GPX1 in liver tissues ↑	WB and RT-PCR	(211)
	Aged mice	Gastrointestinal protection	Protein expression of Nrf2 in intestinal tissues ↑; mRNA expressions of Nrf2, NQO1, HO-1, CAT and SOD2 in intestinal tissues ↑	WB and RT-PCR	(188)
Brown seaweed	Acetaminophen-induced HL-7702 cells	Liver protection	Protein expressions of Nrf2 and nuclear Nrf2 ↑	WB and IF	(182)
*Sargassum kjellmanianum*	H_2_O_2_-induced HUVECs	Anti-diabetic	Protein expressions of Nrf2 and nuclear Nrf2 ↑; protein expression of cytosol Nrf2 ↓	WB and IF	(17)
Antarctic ice microalgae	D-Gal-induced mice	Anti-oxidation	mRNA and protein expressions of Cu/Zn-SOD, Mn-SOD and CAT in liver and spleen tissues ↑; mRNA expressions of Nrf2, HO-1, γ-GCS and NQO1 in liver and spleen tissues ↑; protein expressions of Nrf2, HO-1 and NQO1 in liver and spleen tissues ↑	WB and RT-PCR	(191)
*Padina boryana*	H_2_O_2_-induced Vero cells	Anti-oxidation	Protein expressions of CAT and SOD ↑; protein expression of cytosol Nrf2 ↑; protein expression of cytosol Keap1 ↓	WB	(184)
*Hizikia fusiforme*	H_2_O_2_-treated Vero cells	Anti-oxidation	Protein expressions of Nrf2, CAT and SOD ↑	WB	(44)
*Fucus vesiculosus*	Ca9-22 and CAL27 cells	Anti-cancer	mRNA expressions of Nrf2, TXN and HO-1 ↓	RT-qPCR	(26)
*Coccomyxa Gloeobotrydiformis*	LPS-induced RAW264.7 cells	Anti-inflammation	Protein expressions of HO-1 and nuclear Nrf2 ↑; protein expression of cytosol Nrf2 ↓	WB	(212)
*Padina tetrastromatica*	Isoproterenol-induced rats	Cardioprotection	mRNA expressions of Nrf2 in heart tissues ↑; protein expression of cytosol Nrf2 in heart tissues ↓; protein expression of nuclear Nrf2 in heart tissues ↑	RT-PCR and IF	(189)

Cell experiments have showed that NPs from algae could regulate Nrf2 antioxidant pathway for liver protection (182), lung protection (183), anti-diabetic (17), anti-oxidation (44, 184), and anti-cancer (26). Brown seaweed polysaccharide produced liver protection on acetaminophen-induced HL-7702 cells through increasing protein expressions of Nrf2 and nuclear Nrf2 (182). *Laminaria digitate* polysaccharide generated lung protection against H_2_O_2_-induced MRC-5 cells by up-regulating protein and/or mRNA expressions of nuclear Nrf2, Nrf2, HO-1, NQO1 and GCLC as well as nuclear translocation of Nrf2, and down-regulating Keap1 mRNA (183). *Sargassum kjellmanianum* polysaccharide exhibited anti-diabetic effect on H_2_O_2_-induced HUVECs via elevating protein expressions of Nrf2 and nuclear Nrf2, and declining cytosol Nrf2 protein expression (17). Polysaccharides from *Padina boryana* (184) and *Hizikia fusiforme* (44) showed anti-oxidation action against H_2_O_2_-induced Vero cells via adding protein expressions of cytosol Nrf2, Nrf2, CAT, and SOD, and reducing protein expression of cytosol Keap1. *Fucus vesiculosus* polysaccharide revealed anti-cancer activity on Ca9-22 and CAL27 cells through lowering mRNA expressions of Nrf2, TXN and HO-1 (26).

Animal experiments have showed that NPs from algae could regulate Nrf2 antioxidant pathway for liver protection (31, 97, 185), lung protection (186), kidney protection (97), gastrointestinal protection (187, 188), cardioprotection (189), anti-aging (98, 99), anti-oxidation (190, 191), and immunomodulation (24). NPs from algae possessed liver protection against CTX- (97), CCl_4_- (31), and high-fat diet-induced (185) mice, through increasing protein and/or expressions of p-Nrf2, nuclear Nrf2, cytosol Nrf2, p-Nrf2/Nrf2, HO-1, GCLM, NQO1, CAT, SOD2, Slc7a11, G6pd2, Prdx1, GPX2, and GPX4, and decreasing Keap1 protein expression in liver tissues. *Ecklonia cave* polysaccharide had lung protection on LPS-induced mice by enhancing protein expressions of Nrf2 and HO-1 in lung tissues (186). *Laminaria japonica* polysaccharide exhibited kidney protection against CTX-induced mice via up-regulating protein expressions of Nrf2, HO-1, GCLM, and NQO1 in kidney tissues (97). Meanwhile, this polysaccharide showed anti-aging effect on rotenone-induced rats through rising protein expressions of Nrf2 and PGC-1α in ventral midbrain (98). NPs from algae exerted gastrointestinal protection on heat stress-induced broilers (187) and aged mice (188) by elevating protein and/or mRNA expressions of Nrf2, NQO1, HO-1, CAT, SOD2, GPX1, and GSTT1 in intestinal tissues or duodenum. *Padina tetrastromatica* polysaccharide generated cardioprotection against isoproterenol-induced rats via enhancing protein and/or mRNA expressions of Nrf2 and nuclear Nrf2, and declining cytosol Nrf2 protein expression in heart tissues (189). Those from algae revealed anti-oxidation activity on heat stress-induced *Gallus gallus domesticus* (190) and D-gal-induced mice (191), through aggrandizing mRNA and protein expressions of Nrf2, HO-1, γ-GCS, NQO1, Cu/Zn-SOD, Mn-SOD, SOD2, GSTO1, and CAT in liver and spleen tissues. *Enteromorpha prolifera* polysaccharide reflected immunomodulation against aflatoxin B1-induced broilers by augmenting mRNA and/or protein expressions of SOD1, SOD2, GPX1, GPX3, CAT1, GSTT1, GSTO1, GSTA3, Nrf2, and HO-1 in bursa of fabricius (24).

### Regulation of NPs from fungi

The regulations of NPs on Nrf2 antioxidant pathway from fungi in cell experiments and animal experiments are illustrated in Table 4.

**TABLE 4:** Regulation of NPs from fungi on Nrf2 antioxidant pathway for health-promoting effects.

Polysaccharide source	Experimental model	Health-promoting effects	Regulation on Nrf2 antioxidant pathway	Determination method	References
*Trametes orientalis*	PM_2.5_-induced mice	Lung protection	Protein expressions of Nrf2 and HO-1 in lung tissues ↑	WB	(35)
*Sarcodon aspratus*	H_2_O_2_-induced A549 cells	Lung protection	Protein expression of p-Nrf2 and HO-1 ↑; protein expression of Nrf2 ↓	WB	(105)
	Water immersion and restraint stress-induced rats	Gastrointestinal protection	Protein expression of Keap1 in gastric tissues ↓; Protein expressions of Nrf2, HO-1, NQO1 and NOX4 in gastric tissues ↑	WB and IHC	(106)
*Morchella esculenta*	H_2_O_2_-induced A549 cells	Lung protection	Protein expression of p-Nrf2 and HO-1 ↑; protein expression of Nrf2 ↓	WB	(33)
*Grifola frondosa* fruiting body	LPS/D-GalN-induced mice	Liver protection	Protein expressions of Nrf2, HO-1 and NQO1 in liver tissues ↑; protein expression of Keap1 in liver tissues ↓; mRNA expression of Nrf2 in liver tissues ↑	WB and RT-qPCR	(52)
*Pleurotus geesteranus* fruiting body	Ethanol-induced mice	Liver protection	Protein expressions of Nrf2 and HO-1 in liver tissues ↑	WB and IF	(103)
*Ganoderma lucidum*	High-fat diet-induced diabetic mice	Liver protection	Protein expressions of Nrf2 and HO-1 in liver tissues ↑	WB and IHC	(200)
	H_2_O_2_-induced HSFs	Anti-oxidation	mRNA expression of Keap1 ↓; mRNA expressions of Nrf2, Gstm1, Gstt1, GCLC, GCLM, HO-1 and NQO1 ↑	RT-qPCR	(194)
	Doxorubicin-induced H9c2 cells	Cardioprotection	Protein expressions of Nrf2 and HO-1 ↑	WB	(193)
*Termitomyces albuminosus* mycelium	CCl_4_-induced mice	Liver protection	mRNA expressions of Nrf2 and HO-1 in liver tissues ↑	RT-qPCR	(107)
*Inonotus obliquus*	*Toxoplasma gondii*-induced mice	Liver protection	Protein expressions of HO-1 and nuclear Nrf2 in liver tissues ↑	WB	(104)
		Improving reproductive function	Protein expressions of HO-1, NQO1 and nuclear Nrf2 in testicular tissues ↑		(27)
	AD model APP/PS1 mice	Anti-aging	Protein expression of Keap1 in brain tissues ↓; protein expressions of Nrf2, SOD-1, HO-1 and GCLC in brain tissues ↑	WB	(196)
	L-Glu-induced HT22 cells	Anti-aging	Protein expression of Keap1 ↓; protein expressions of Nrf2, SOD-1, HO-1 and GCLC ↑		
*Antrodia camphorata*	LPS/D-GalN-induced mice	Liver protection	Protein expressions of Keap1, Nrf2 and γ-GCS in liver tissues ↑	WB	(192)
	LPS-induced Kupffer cells		Protein expressions of Keap1, Nrf2 and γ-GCS ↑	WB and IF	
*Cordyceps militaris*	Pb^2+^-induced mice	Kidney protection	Protein expressions of Keap1, Nrf2, HO-1 and NQO1 in kidney tissues ↑	WB	(39)
*Amanita caesarea*	L-Glu induced HT22 cells	Anti-aging	Protein expressions of cytosol Nrf2 ↓; protein expressions of nuclear Nrf2 ↑	WB	(108)
	AD model APP/PS1 mice		Protein expressions of Nrf2 and HO-1 in hippocampus ↑; protein expressions of Keap1 ↓		(109)
*Hericium erinaceus* mycelium	AD model APP/PS1 mice	Anti-aging	Protein expressions of Nrf2 and HO-1 hippocampus ↑; protein expressions of Keap1 in hippocampus ↓	WB	(110)
*Tremella fuciformis*	UVA-induced HDF cells	Anti-aging	Protein expressions of NQO1 and nuclear Nrf2 ↑; protein expression Keap1 and cytosol Nrf2 ↓; mRNA expressions of Nrf2, HO-1 and NQO1 ↑; mRNA expression of Keap1 ↓	ELISA and RT-qPCR	(197)
*Suillellus luridus*	STZ-induced mice	Anti-diabetic	mRNA and protein expressions of Nrf2 and HO-1 in liver tissues ↑	WB and RT-PCR	(111)
*Paecilomyces hepialid* mycelium	db/db mice	Anti-diabetic	Protein expressions of Nrf2, HO-1 and CAT in kidney tissues ↑	WB	(101)
*Lentinus edodes* mycelium	High glucose-induced MIN6 cells	Anti-diabetic	Protein expression of nuclear Nrf2 ↑	WB	(198)
	High glucose-induced INS-1 cells				(102)
*Saccharomyces cerevisiae*	LPS-induced RAW264.7 cells	Anti-oxidation	HO activity ↑; protein expressions of Nrf2 and HO-1 ↑	Assay kits, WB and IF	(195)
*Lachnum* sp.	HepG2 cells	Anti-cancer	Protein expression of Nrf2 ↓; protein expression of Keap1, HO-1, NQO1, GST1, SOD2, GPX and GCLM ↑	WB and IF	(199)
*Antrodia cinnamomea*	CTX-induced mice	Immunomodulation	Protein expression of Keap1 in spleen and thymus ↓; protein expression of Nrf2, HO-1, SOD2 and CAT in spleen and thymus ↑	WB	(202)
*Sarcodon imbricatus*	CTX-induced mice	Immunomodulation	Protein expressions of Nrf2, HO-1, SOD1, SOD2, CAT and NQO1 in spleen ↑	WB	(203)
*Poria cocos*	ox-LDL-induced VSMCs	Anti-atherosclerosis	Protein expressions of HO-1 and nuclear Nrf2 ↑; protein expressions of cytosol Nrf2 ↓	WB	(29)
	5-Fu-treated CT26 tumor-bearing mice	Gastrointestinal protection	Protein expressions of Nrf2 in colon tissues ↑	IHC	(201)
*Ganoderma atrum*	LPS-induced Caco-2/RAW264.7 co-culture inflammation	Gastrointestinal protection	Protein expressions of Keap1 and Nrf2 ↑	WB	(113)

Cell experiments have showed that NPs from fungi could regulate Nrf2 antioxidant pathway for liver protection (192), lung protection (33, 105), cardioprotection (193), gastrointestinal protection (113), anti-oxidation (194, 195), anti-aging (108, 196, 197), anti-diabetic (102, 198), anti-cancer (199), and anti-atherosclerosis (29). *Antrodia camphorate* polysaccharide exhibited liver protection on LPS-induced Kupffer cells by increasing protein expressions of Keap1, Nrf2, and γ-GCS (192). Polysaccharides from *Sarcodon aspratus* (105) and *Morchella esculenta* (33) exerted lung protection against H_2_O_2_-induced A549 cells via adding protein expressions of p-Nrf2 and HO-1, and reducing Nrf2 protein expression. *Ganoderma lucidum* polysaccharide showed cardioprotection on doxorubicin-induced H9c2 cells through rising protein expressions of Nrf2 and HO-1 (193). *Ganoderma atrum* polysaccharide reflected gastrointestinal protection in LPS-induced Caco-2/RAW264.7 co-culture inflammation model by up-regulating protein expressions of Keap1 and Nrf2 (113). NPs from fungi had anti-oxidation activity on H_2_O_2_-induced HSFs (194) and LPS-induced RAW264.7 cells (195) through augmenting protein and/or mRNA expressions of Nrf2, Gstm1, Gstt1, GCLC, GCLM, HO-1, and NQO1, and reducing Keap1 mRNA expression. Those from fungi displayed anti-aging effect against L-Glu-induced HT22 cells (108, 196) and UVA-induced HDF cells (197) via aggrandizing protein and/or mRNA expressions of nuclear Nrf2, Nrf2, SOD1, HO-1, NQO1, and GCLC, and lowering protein and/or mRNA expressions of Keap1 and cytosol Nrf2. *Lentinus edodes* mycelium polysaccharide had anti-diabetic action against high glucose-induced MIN6 or INS-1 cells, which was related to increment of nuclear Nrf2 protein expression (102, 198). *Lachnum* sp. polysaccharide possessed anti-cancer activity on HepG2 cells involved with reduction of Nrf2 protein expression, and enhancement of protein expression of Keap1, HO-1, NQO1, GST1, SOD2, GPX, and GCLM (199). *Poria cocos* polysaccharide caused anti-atherosclerosis effect on ox-LDL-induced VSMCs by rising protein expressions of HO-1 and nuclear Nrf2, and declining cytosol Nrf2 protein expression (29).

Animal experiments have demonstrated that NPs from fungi could regulate Nrf2 antioxidant pathway for liver protection (52, 103, 104, 107, 192, 200), lung protection (35), kidney protection (39), gastrointestinal protection (106, 201), anti-aging (109, 110, 196), anti-diabetic (101, 111), improving reproductive function (27), and immunomodulation (202, 203). NPs from fungi exhibited liver protection against LPS/D-GalN- (52, 192), ethanol- (103), high-fat diet- (200), CCl_4_- (107), and *Toxoplasma gondii*-induced (104) mice, through increment of protein and/or mRNA expressions of nuclear Nrf2, Nrf2, HO-1, NQO1, and γ-GCS, and modulation of Keap1 protein expression in liver tissues. *Trametes orientalis* polysaccharide exerted lung protection on PM_2.5_-induced mice by increasing protein expressions of Nrf2 and HO-1 in lung tissues (35). *Cordyceps militaris* polysaccharide showed kidney protection against Pb^2+^-induced mice via enhancing protein expressions of Keap1, Nrf2, HO-1, and NQO1 in kidney tissues (39). NPs from fungi possessed gastrointestinal protection on water immersion and restraint stress-induced rats (106) and 5-Fu-treated CT26 tumor-bearing mice (201), through elevating protein expressions of Nrf2, HO-1, NQO1, and NOX4, and reducing Keap1 protein expression in gastric or colon tissues. Polysaccharides from *Inonotus obliquus* (196), *Amanita caesarea* (109) and *Hericium erinaceus* mycelium (110) revealed anti-aging activity on AD model APP/PS1 mice via elevating protein expressions of Nrf2, SOD-1, HO-1, and GCLC, and reducing Keap1 protein expression in brain tissues or hippocampus. NPs from fungi appeared anti-diabetic function against STZ-induced (111) and db/db mice (101) by promoting mRNA and protein expressions of Nrf2, HO-1 and CAT in liver or kidney tissues. *Inonotus obliquus* polysaccharide improved reproductive function of *Toxoplasma gondii*-induced mice through up-regulating protein expressions of HO-1, NQO1 and nuclear Nrf2 in testicular tissues (27). Polysaccharides from *Antrodia cinnamomea* (202) and *Sarcodon imbricatus* (203) displayed immunomodulation against CTX-induced mice by increasing protein expressions of Nrf2, HO-1, SOD1, SOD2, CAT, and NQO1, and decreasing Keap1 protein expression in spleen or thymus.

### Regulation of NPs from animals and bacteria

Polysaccharides from animals (*Ostrea plicatula* Gmelin, *Holothuria leucospilota*, *Acaudina leucoprocta*, *Sepia esculenta* ink, and *Ostrea rivularis*) as well as chitosan could regulate Nrf2 antioxidant pathway for liver protection (114, 204), anti-oxidation (115), improving reproductive function (43, 205, 206), and gastrointestinal protection (207), as summarized in Table 5. Cell experiment indicated that *Acaudina leucoprocta* polysaccharide exerted anti-oxidation effect on H_2_O_2_-induced RAW264.7 cells by increasing mRNA and/or protein expressions of Nrf2, SOD1, and GPX1, and decreasing Keap1 protein expression (115). In animal experiments, polysaccharides from *Ostrea plicatula* Gmelin (204) and *Holothuria leucospilota* (114) exhibited liver protection against CTX-induced mice and type 2 diabetic rats respectively, involving with increment of protein and/or mRNA expressions of Nrf2, HO-1, and NQO1 in liver tissues. NPs from animals improved reproductive function against CTX-induced mice (43, 205, 206) through elevating protein and/or mRNA expressions of Nrf2, HO-1, and NQO1, and modulating Keap1 protein expression in ovarian or testis. Chitosan displayed gastrointestinal protection on piglets by adding protein and/or mRNA expressions of GPX1, GPX2, SOD1, SOD2, CAT, Nrf2, NQO1, and HO-1, and declining Keap1 protein expression in ileum (207).

**TABLE 5:** Regulation of NPs from animals and bacteria on Nrf2 antioxidant pathway for health-promoting effects.

Polysaccharide source	Experimental model	Health-promoting effects	Regulation on Nrf2 antioxidant pathway	Determination method	References
*Ostrea plicatula* Gmelin	CTX-induced mice	Liver protection	Protein expressions of Nrf2, HO-1 and NQO1 in liver tissues ↑	WB	(204)
*Holothuria leucospilota*	Type 2 diabetic rats	Liver protection	Protein and mRNA expressions of Nrf2 and HO-1 in liver tissues ↑	RT-qPCR and IHC	(114)
*Acaudina leucoprocta*	H_2_O_2_-induced RAW264.7 cells	Anti-oxidation	mRNA expressions of SOD1 and GPX1 ↑; protein expression of Keap1 ↓; protein expression of Nrf2 ↑	WB and RT-PCR	(115)
*Sepia esculenta* ink	CTX-induced mice	Improving reproductive function	Protein expressions of Nrf2, HO-1 and NQO1 in ovarian ↑; protein expression of Keap1 in ovarian ↓	WB	(205)
			Protein expressions of Keap1, Nrf2, HO-1 and NQO1 in testicular tissues ↑	WB	(206)
*Ostrea rivularis*	CTX-induced mice	Improving reproductive function	mRNA expressions of Nrf2, HO-1 and NQO1 in testis ↑; protein expressions of Keap1, Nrf2 and HO-1 in testis ↑	WB and RT-PCR	(43)
Chitosan	piglets	Gastrointestinal protection	Protein expression of Keap1 in ileum ↓; protein expression of Nrf2 in ileum ↑; mRNA expressions of GPX1, GPX2, SOD1, SOD2, CAT, Nrf2, NQO1 and HO-1 in ileum ↑	WB and RT-PCR	(207)
*Bacillus megaterium*	H_2_O_2_-induced WI38 cells	Lung protection	Cytosol: protein expressions of Keap1 and Nrf2 ↑; Nuclear: protein expressions of Keap1 and Nrf2 ↓; Nuclear translocation of Nrf2 ↓	WB and IF	(38)
	A549 cells	Anti-cancer	Protein expressions of cytosol Keap1 and Nrf2 ↓; protein expressions of nuclear Keap1 and Nrf2 ↑	WB and IF	(53)

Polysaccharides from *Bacillus megaterium* could regulate Nrf2 antioxidant pathway for lung protection (38) and anti-cancer (53), as listed in Table 5. Cell experiments have demonstrated that this polysaccharide exerted lung protection on H_2_O_2_-induced WI38 cells by enhancing protein expressions of cytosol Keap1 and cytosol Nrf2, and suppressing protein expressions of nuclear Keap1 and Nrf2 as well as nuclear translocation of Nrf2 (38). Meanwhile, the polysaccharide exhibited anti-cancer effect on A549 cells through increasing protein expressions of cytosol Keap1 and Nrf2, and decreasing protein expressions of nuclear Keap1 and Nrf2 (53).

**FIGURE 2:**
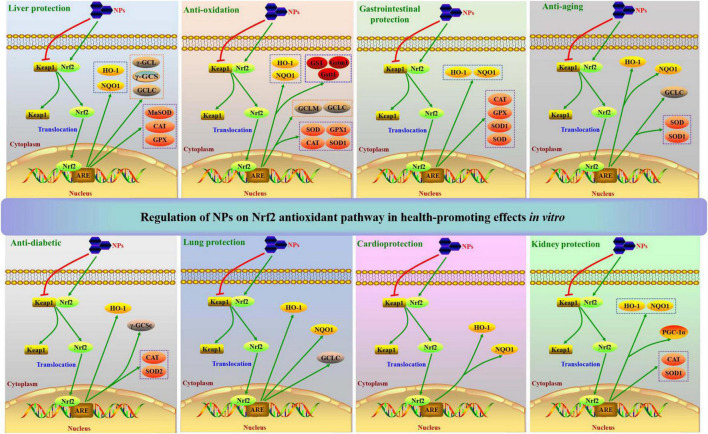
Regulations of NPs on Nrf2 antioxidant pathway in health-promoting effects *in vitro*.

**FIGURE 3:**
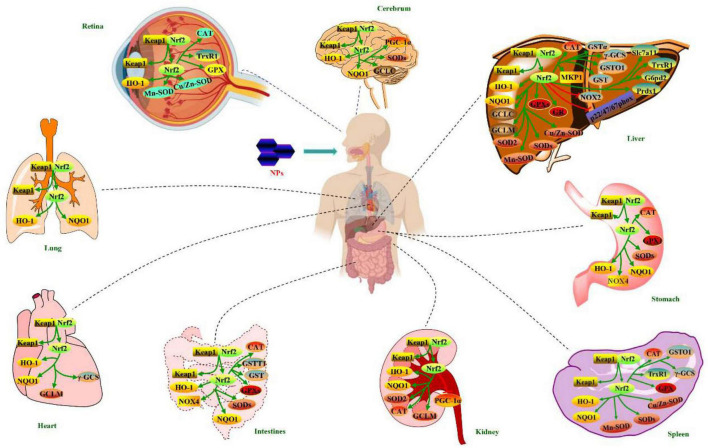
Regulations of NPs on Nrf2 antioxidant pathway in health-promoting effects *in vivo*.

With above analyses, regulations of NPs on Nrf2 antioxidant pathway in health-promoting effects *in vitro* and *in vivo* can be summarized in Figures 2, 3, respectively.

## Structure-activity relationship of NPs for health-promoting effects by regulating Nrf2 antioxidant pathway

Structure-activity relationship of NPs for health-promoting effects by regulating Nrf2 antioxidant pathway is unclear. However, the influences of *M*_*w*_, functional group, monosaccharide composition and side chains on the efficacies of NPs in regulating Nrf2 antioxidant pathway could be preliminarily discussed.

### Influence of *M*_*w*_

There might be two different standpoints concerning the influence of *M*_*w*_ on the regulation of NPs to Nrf2 antioxidant pathway. One standpoint is that polysaccharide with higher *M*_*w*_ generated stronger regulation on Nrf2 antioxidant pathway *in vitro* and *in vivo*. Polysaccharide (AZP-1a) with higher *M*_*w*_ (34.1 kDa) from *Anoectochilus zhejiangensis* exhibited better protection on CCl_4_-treated HepG2 cells than that (AZP-1d) with lower *M*_*w*_ (4.568 kDa). And, the former enhanced more protein expressions of Nrf2, HO-1 and NQO1 in HepG2 cells (70). Jing et al. (85) have obtained five fractions (PS-1, 14.561 kDa; PS-2, 19.783 kDa; PS-3, 4.973 kDa; PS-4, 15.928 kDa; PS-5, 7.046 kDa) from *Athyrium Multidentatum* and evaluated theirs cytoprotective activities against H_2_O_2_-induced HUVECs. Results indicated that the two higher *M*_*w*_ fractions (PS-2 and PS-4) possessed relatively higher cytoprotections and caused more mRNA expressions of Nrf2 and HO-1 than other three lower *M*_*w*_ fractions. Polysaccharide (PNP40c-1) with higher *M*_*w*_ (206 kDa) from pine nut exerted stronger hepatoprotection against CCl_4_-induced liver damage in mice and up-regulated more mRNA expressions of Nrf2 and HO-1 in the liver than that (PNP80b-2) with lower *M*_*w*_ (23.0 kDa) (87, 90). Two purified polysaccharides (RGP-1-A and RGP-2-A) were obtained from *Rehmannia glutinosa* after decolorization using AB-8 macroporous resin and H_2_O_2_ respectively, and their *M*_*w*_ values were 18.964 and 3.305 kDa. RGP-1-A showed significantly higher antioxidant capacity on H_2_O_2_-induced IPEC-1 cells and caused more up-regulation on mRNA expressions of Nrf2, HO-1 and NQO1 and less Keap1 mRNA expression (208).

Another standpoint is that polysaccharide with lower *M*_*w*_ caused stronger regulation on Nrf2 antioxidant pathway *in vitro* and *in vivo*. Polysaccharide (TOP-2) with smaller *M*_*w*_ (<1 kDa) from *Taraxacum officinale* elevated more protein expressions of Nrf2 and HO-1 than that (TOP-1) with larger *M*_*w*_ (1–9.3 kDa) in LPS-induced RAW264.7 cells, although TOP-2 and TOP-1 had no significance in protecting RAW264.7 cells (132). Polysaccharide (DRP1) with lower *M*_*w*_ (5.695 kDa) from Dandelion root reflected better hepatoprotection on CCl_4_-induced liver injury in mice than that (DRP2) with higher *M*_*w*_ (8.882 kDa). Meanwhile, DRP1 increased relatively more mRNA expressions of Nrf2 and NQO1 while decreased more mRNA expression of Keap1 in the liver than DRP2 (57). Polysaccharide (FWBP, 21.19 kDa) from fermented wheat bran has been shown to be more effectiveness in positively regulating gut antioxidant-associated gene expression and gut microbiota in zebrafish than that (WBP, 52.03 kDa) from wheat bran. At the same time, FWBP produced more mRNA expressions of CAT, GST, and Nrf2 along with less GPX-3 mRNA expression than than WBP in zebrafish (162). Two different polysaccharides (CPSP-1, 13.1 kDa; CTSP-1, 23.0 kDa) have been obtained from stems of *Codonopsis pilosula* and *Codonopsis tangshen*, respectively (66). CPSP-1 showed higher protective effect on H_2_O_2_-induced IPEC-J2 cells and had a better promotion on GPXs and SOD1 expressions than CTSP-1. Meanwhile, a polysaccharide (CPP-1) with *M*_*w*_ of 21.0 kDa from *Codonopsis pilosula* roots showed stronger protection on H_2_O_2_-induced IPEC-J2 cells and regulation on Nrf2 antioxidant pathway than that (CTP-1) with *M*_*w*_ of 29.5 kDa from *Codonopsis tangshen* roots (55).

However, polysaccharide with moderate *M*_*w*_ might be more beneficial to regulate Nrf2 antioxidant pathway. For example, Han et al. (60) have investigated the repair effects of three *Astragalus* polysaccharides (APS0, APS1, and APS2) with different *M*_*w*_ (11.03, 4.72, and 2.61 KDa) against oxalate-induced HK-2 cells. The findings displayed that APS1 with the moderate *M*_*w*_ provided the strongest repair effect and increased the most protein expressions of Keap1, Nrf2, SOD1, and CAT.

### Influence of functional group

Selenization, sulfuration, and acetylation modifications could improve the regulation of NPs on Nrf2 antioxidant pathway, owing to new functional groups have been brought in. Selenizing *Codonopsis pilosula* polysaccharides (sCPPS_5_) caused significantly stronger protective effect on H_2_O_2_-induced RAW264.7 cells and more increases in protein expressions of Nrf2, HO-1, NQO1, GCLM, and GCLC and declination in Keap1 protein expression than unmodified polysaccharide (CPPS) (131). Selenizing *Astragalus* polysaccharides (sAPS) exhibited markedly higher protection against CCl_4_-induced liver injury in rats and up-regulated more mRNA expression levels of GPX1, SOD1 and Nrf2 in the liver than the native one (APS) (150). On the other hand, sulfated *Cyclocarya paliurus* polysaccharide (S-CPP_0.05_) showed stronger antioxidant activity to H_2_O_2_-induced DCs and generated more increment in Nrf2 protein expression and reduction in Keap1 protein expression in DCs, as compared with the native one (CPP_0.05_) (96). At the dosages of 100 and 200 mg/kg, sulfated *Codonopsis* polysaccharide (SCP) produced better hepatoprotective effect on liver in ethanol-induced mice and more decreases in mRNA expressions of Nrf2 and Keap1 than the native one (CP) in the liver (164). Otherwise, acetylated *Cyclocarya paliurus* polysaccharide (Ac-CPP_0.1_) generated higher cytoprotection on H_2_O_2_-induced DCs and improved more mRNA expressions of SOD1, GPX1, CAT, HO-1, and NQO1 than the native one (CPP_0.1_) (72). Acetylated *Stropharia rugoso-annulata* polysaccharides (ASRP) exhibited better action in alleviating non-alcoholic fatty liver in HFD-induced mice and caused more HO-1 protein expression and less Keap1 protein expression in liver tissues (209).

### Influence of monosaccharide composition

Natural polysaccharides with higher GalA or GlcA may cause better regulation effect on Nrf2 antioxidant pathway. Two polysaccharides (CPSP-1 and CTSP-1) gained from stems of *Codonopsis pilosula* and *Codonopsis tangshen* were determined to contain GalA of 70.1 and 61.3%, respectively. The former was proven to have better protective action on H_2_O_2_-induced IPEC-J2 cells and regulation effect on Nrf2 antioxidant pathway (66). Five fractions (PS-1, PS-2, PS-3, PS-4, and PS-4) from *Athyrium multidentatum* were characterized to contain GlcA content with an order as PS-1 < PS-5 < PS-4 < PS-2 < PS-3 (85). PS-1 showed the lowest cytoprotection on H_2_O_2_-induced HUVECs cells and regulation on mRNA expressions of Nrf2 and HO-1. Two purified polysaccharides (RGP-1-A and RGP-2-A) obtained from *Rehmannia glutinosa* were determined to have GalA contents of 19.02 and 1.1%. RGP-1-A showed significantly better cytoprotection on H_2_O_2_-induced IPEC-1 cells and caused observably more increments in mRNA expressions of Nrf2, HO-1 and NQO1 and reduction in Keap1 mRNA expression (208).

On the other hand, higher contents of Ara, Gal, and Rha may have greater regulation effect on Nrf2 antioxidant pathway. The polysaccharides (CPP-1 and CTP-1) from roots of *Codonopsis pilosula* and *Codonopsis tangshen* contained Ara+Gal+Rha contents of 41.1 and 39%, respectively. CPP-1 revealed relatively protection on H_2_O_2_-induced IPEC-J2 cells and greater regulation on Nrf2 antioxidant pathway (55). Meanwhile, the above-mentioned PS-1 with smallest Ara+Gal+Rha contents showed the lowest cytoprotection on H_2_O_2_-induced HUVECs cells and regulation on mRNA expressions of Nrf2 and HO-1, as compared with PS-2, PS-3, PS-4, and PS-5 (85).

### Influence of side chains

Shorter AG side chains of NPs can be more effective in promoting Nrf2 antioxidant pathway. A polysaccharide (CPSP-1) with AG-II chains acquired from *Codonopsis pilosula* stems showed stronger protective effect on H_2_O_2_-induced IPEC-J2 cells and promotion on Nrf2 antioxidant pathway than that (CTSP-1) with AG-I and AG-II chains from *Codonopsis tangshen* stems (66). Moreover, CPP-1 with shorter AG-I chains from *Codonopsis pilosula* roots revealed better protection on H_2_O_2_-induced IPEC-J2 cells and regulation on Nrf2 antioxidant pathway than CTP-1 with longer AG-I chains from *Codonopsis tangshen* roots (55).

## Conclusions and prospects

This review summarizes that NPs from natural sources can regulate Nrf2 antioxidant pathway to exert a wide spectrum of health-promoting effects *in vitro* and *in vivo*, such as liver protection, kidney protection, lung protection, neuroprotection, cardioprotection, gastrointestinal protection, anti-oxidation, anti-diabetic, anti-aging, anti-inflammation, anti-radiation, anti-depression, anti-cancer, anti-atherosclerosis, immunomodulation, and improving reproductive function. Moreover, some factors like Keap1, Nrf2, HO-1, NQO1, GCLC, GCLM, γ-GCL, γ-GCS, γ-GCSc, Mn-SOD, SODs, GPXs, CAT, GST, Gstm1, Gstt1, and PGC-1α in Nrf2 antioxidant pathway are modulated in the frequently seen *in vitro* health-promoting effects (liver protection, kidney protection, lung protection, cardioprotection, gastrointestinal protection, anti-oxidation, anti-diabetic and anti-aging) of NPs (Figure 2). Meanwhile, Keap1, Nrf2, HO-1, NQO1, GCLC, GCLM, γ-GCS, Cu/Zn-SOD, Mn-SOD, SODs, GPXs, GR, CAT, GSTs, NOX2, NOX4, TrxR1, Slc7a11, G6pd2, Prdx1, PGC-1α, MKP1, and p22/47/67phox are regulated in these *in vivo* health-promoting effects (Figure 3). On the other hand, NPs having regulation on Nrf2 antioxidant pathway can be widely acquired by water extraction and column chromatography methods. *M*_*w*_ of obtained NPs ranges from 1.206 to 3440 kDa, and Fuc, Rha, Ara, Gal, Glc, and/or Man are widely discovered in them. A variety of structures, like pectin, arabinogalactan, 2-*O*-acetylglucomannan, glucan, and glucogalactan, have been determined in NPs which having regulation on Nrf2 antioxidant pathway. NPs are frequently composed of T-Ara*f*-(1→, →5)-Ara*f*-(1→, →3)-Gal*p*-(1→, →6)-Gal*p*-(1→, →3,4)-Gal*p*-(1→, →3,6)-Gal*p*-(1→, T-Glc*p*-(1→, →3)-Glc*p*-(1→, →4)-Glc*p*-(1→, →6)-Glc*p*-(1→ and →4)-GalA*p*-(1→ residues. And →4)-Glc*p*-(1→, →6)-Glc*p*-(1→, →3)-Gal*p*-(1→ and →4)-D-Man*p*-(1→ residues are commonly distributed in their backbones. Noteworthily, structural features of NPs are different owing to different methods and protocols used in extraction and purification processes, thereby structural features included *M*_*w*_, functional group, monosaccharide composition and side chains have influences on the efficacies of NPs in regulating Nrf2 antioxidant pathway.

Although many studies have disclosed the regulation of NPs on Nrf2 antioxidant pathway, there are still some problems should be explored in future: (i) compared with NPs from herbs and woody plants, less researches have been conducted to the regulative effects of NPs from algae, fungi, animals, and bacteria on Nrf2 antioxidant pathway; (ii) existing evidences are inadequate to establish structure-activity relationship for regulation of NPs on Nrf2 antioxidant pathway in their health-promoting effects; (iii) clinical research on the regulation of NPs on Nrf2 antioxidant pathway is scarce, and regulation of NPs on Nrf2 antioxidant pathway is rarely reported in some health-promoting effects; (iv) Nrf2 antioxidant pathway is activated by NPs in most cases, whilst it is inhibited by NPs in several health-promoting effects like anti-cancer. However, there is few information concerning the classification of NPs as activators and inhibitors; (v) as shown in Tables 1–5, regulation of NPs on Nrf2 antioxidant pathway has been determined by WB, RT-PCR, RT-qPCR, IHC, IF, ChIP, EMSA, and ELISA as well as assay kits. However, Nrf2 antioxidant pathway is a complex network and it has some relations with other pathways. Thus, proteomics, transcriptomics and other methods can be used to explore the regulation of NPs on Nrf2 antioxidant pathway; (vi) there are many genes like PI3K, JNK, ERK, and AKT can regulate Nrf2 antioxidant pathway (10), the effects of NPs on these genes should also be explored; (vii) which procedure is more suitable for preparing NPs with regulation on Nrf2 antioxidant pathway, and which structure has the stronger regulation, cannot be concluded.

## Author contributions

J-HL and JL: investigation, writing—original draft, and visualization. Z-CS, X-FL, and Y-FW: investigation. E-SG: writing—review and editing. QZ: project administration and funding acquisition. X-YW: writing—review and editing, supervision, project administration, and funding acquisition. All authors contributed to the article and approved the submitted version.
