# Supplementary material for: Advances in health-promoting effects of natural polysaccharides: Regulation on Nrf2 antioxidant pathway
Source: Front Nutr. 2023 Feb 16;10:1102146. doi: 10.3389/fnut.2023.1102146 (PMC9978827; doi:10.3389/fnut.2023.1102146)
Supplement: Supplementary file 1 [file Table_1.docx]

**Table S1** Structural characteristics of NPs which exert health-promoting effects by regulating Nrf2 antioxidant pathway

| Sources | Code | Extraction and/or purification methods | *M*_w_ (kDa) | Monosaccharide composition | Glycosidic bond types | Backbone | Side chains | Health-promoting effects | References |
| --- | --- | --- | --- | --- | --- | --- | --- | --- | --- |
| **Herb** | | | | | | | | | |
| *Dendrobium officinale* | DOPS | Water extraction, DEAE cellulose-52, Sephacryl S-400 and Sephacryl S-200 chromatography | 393.8 | Man: Glc: Ara = 5.55: 1: 0.12 (molar ratio) | T-Man*p*-(1→: T-Ara*f*-(1→: →3)-Glc*p*-(1→: →3)-Man*p*-(1→: →4)-Man*p*-(1→: →4)-Glc*p*-(1→: →4,6)-Man*p*-(1→: →4,6)-Glc*p*-(1→ = 1.2: 1: 0.9: 3.5: 26.1: 5.8: 1.4: 0.6 (molar ratio) | →4)-β-D-Man*p*-(1→ and →4)-β-D-Glc*p*-(1→ | →3)-β-D-Glc*p*-(1→ and →3)-β-D-Man*p*-(1→ at O-6, and acetyl groups at O-2 | Lung protection | (20, 63) |
|  |  |  |  |  |  |  |  | Liver protection | (67, 68) |
|  |  |  |  |  |  |  |  | Improving learning and memory impairment |  |
|  | DOP | Water extraction | 8.551 | NA | NA | NA | NA | Liver protection | (121) |
|  | M-DOP | Water extraction and ultrasonic treatment | 75.41 | Ara: Gal: Glc: Man: Rha = 0.38: 0.40: 1.00: 0.15: 0.02 (molar ratio) | NA | NA | NA | Anti-aging | (18) |
| *Dendrobium fimbriatum* | cDFPW1 | Water extraction, DEAE cellulose-52 and Sephadex G-75 chromatography | 40 | Man and Glc | T-Man*p*-(1→: →4)-Glc*p*-(1→: →4)-Man*p*-(1→ and →4,6)-Glc*p*-(1→ = 1.3: 7.1: 1.0: 1.9 (molar ratio) | NA | NA | Gastrointestinal protection | (16) |
| Dandelion root | DRP1 | Water extraction, DEAE cellulose-52 and Sephacryl S-200 chromatography | 5.695 | Glc: Gal: Ara = 78.11: 3.07: 18.82 (molar percentage) | →4)-Ara*f*-(1→: T-Gal*p*-(1→: →6)-Glc*p*-(1→: →3,4)-Glc*p*-(1→: →2,4)-Glc*p*-(1→: →2,6)-Glc*p*-(1→ = 8.95: 2.15: 15.48: 10.43: 8.67: 3.86 (molar ratio) | →6)-α-D-Glc*p*-(1→ and →3,4)-α-D-Glc*p*-(1→ | NA | Liver protection | (57) |
|  | DRP2 |  | 8.882 | Rha: GlcA: Glc: Gal: Ara = 4.40: 17.84: 42.59: 13.34: 21.84 (molar percentage) | T-Ara*f*-(1→: →2)-Rha*p*-(1→: →3,6)-Glc*p*-(1→: T-Glc*p*-(1→: →2,4)-Glc*p*-(1→: →3)-Gal*p*-(1→: →2,6)-Gal*p*-(1→ = 4.96: 0.34: 1.71: 4.58: 1.37: 0.52: 2.15 (molar ratio) | T-α-D-Ara*f*-(1→ and T-α-D-Glc*p*-(1→ | NA |  | (57) |
| *iticum aestivum* sprouts | TASP | Water extraction and DEAE Sephadex A-25 chromatography | NA | Glc: Man: Gal = 1.4: 1.0: 2.1 (molar ratio) | NA | NA | NA | Liver protection | (122) |
| *Panax notoginseng* | PNPS-0.5M | Water extraction, DEAE-Sepharose Fast Flow and Superdex-200 chromatography | 2617 | Rha: Ara: Xyl: Man: Glc: Gal = 3.8: 4.5: 2.0: 1.6: 89.8 (molar ratio) | T-Ara*f*-(1→: T-Rha*p*-(1→: →5)-Ara*f*-(1→: →4)-GalA*p*-(1→: →3,6)-Gal*p*-(1→ = 6.23: 4.17: 4.85: 79.58: 5.17 (molar ratio) | →4)-GalA*p*-(1→ | T-Ara*f*-(1→, T-Rha*p*-(1→ and →5)-Ara*f*-(1→ | Liver protection | (54) |
| *Dicliptera chinensis* | DCP | Ethanol precipitation | 2.273 | Glc: Gal: Ara: Rha: Man = 3.20: 2.54: 1.69: 1.58: 1.00 (molar ratio) | NA | NA | NA | Liver protection | (41) |
| *Echinacea purpurea* | EPP80 | Ultrasonic extraction and stepwise ethanol precipitation | 11.82 | Ara: Gal: Glc: Man: GalA: GlcA = 13.42: 25.12: 10.92: 8.59: 2.07: 0.82 (molar ratio) | NA | NA | NA | Liver protection | (36) |
|  | EPPS-3 | Water extraction, DEAE cellulose Purose 6 Fast Flow and Sephadex G100 chromatography | 13.0 | Glc: Gal: Ara: GalA: GlcA = 34: 17: 30: 10: 4 (molar ratio) | NA | NA | NA | Liver protection | (120) |
| *Codonopsis lanceolata* | CLPS | Water extraction | 65.6 | Rha: Ara: Xyl: Man: Gal: Glc: GalA: GlcA = 0.17: 1: 0.12: 0.05: 0.26: 2.32: 0.19: 0.95 (molar ratio) | NA | NA | NA | Anti-insulin resistance | (58) |
| Pumpkin | Polysaccharide | Water extraction, DEAE-cellulose anion-exchange and Sephadex G-100 chromatography | 607.6 | Man: Rib: GlcN: GlcA: GalA: Glc: GalN: Xyl: Fuc = 142.92: 42.89: 1.03: 17.83: 2.6: 125.75: 0.85: 112.34: 73.25 (mass ratio) | NA | NA | NA | Anti-diabetic | (56) |
| *Abelmoschus esculentus* | OP | Water extraction, DEAE-Sepharose Fast Flow and Sepharose CL-6B | 626 | Man: Rha: GlcA: GalA: Gal: Ara = 3.4: 3.76: 24.19: 6.27: 8.73: 3.13 (molar ratio) | NA | NA | NA | Anti-diabetic | (123, 124) |
| *Hosta ventricosa* | HVRPp-1 | Water extraction, DEAE cellulose-52 and Sephadex G-100 chromatography | 11.6 | GlcA: Man: Glc = 8.4: 13.5: 1 (molar ratio) | NA | NA | NA | Antioxidant | (125) |
| Fermented wheat bran | FWBP | Water extraction and DEAE cellulose-52 chromatography | 21.19 | Man: Rib: Rha: GlcA: GalA: Glc: Gal: Xyl: Ara: Fuc = 2.65: 0.12: 1.2: 0.13: 0.04: 17.43: 2.13: 43.32: 32.66: 0.32 (molar ratio) | NA | NA | NA | Antioxidant | (76, 77) |
| *Thymus quinquecostatus* | DJP70-1 | Water extraction, stepwise ethanol precipitation and DEAE-cellulose 52 chromatography | ＞2457 | Man: Rib: Rha: GlcA: GalA, Glc: Gal: Xyl: Ara = 16.03: 1.00: 4.14: 1.73: 4.54: 5.48: 19.87: 1.01: 14.70 (molar ratio) | →4)-Rib*p*-(1→: →2)-Ara*f*-(1→: →5)-Ara*f*-(1→: →3, 6)-Man*p*-(1→: →4)-Glc*p*-(1→: T-Glc*p*-(1→: →4, 6)-Man*p*-(1→: →6)-Gal*p*-(1→: →4)-Man*p*-(1→: →6)-Glcp-(1→: →3,6)-Gal*p*-(1→: →6)-Man*p*-(1→ = 1.33: 0.43: 0.84: 2.45: 0.20: 1.89: 0.93: 1.56: 0.35: 1.90: 3.19: 0.72 (molar ratio) | →5)-α-L-Ara*f*-(1→ and →6)-β-D-Man*p*-(1→ | NA | Anti-oxidation | (47) |
| Wheat germ | WGP-1 | Water extraction, DEAE-cellulose 52 and Sephacryl S-500 chromatography | 195 | Rha: Ara: Xyl: Man: Gal: Glc = 5.93: 28.45: 9.67: 5.35: 8.89: 41.70 (mass percentage) | NA | NA | NA | Anti-oxidation | (88) |
| *Apiosa mericana* Medikus | ATP-1 | Water extraction and DEAE cellulose-52 chromatography | 12.16 | Glc: Ara: Gal: GalA = 96.23: 2.51: 0.59: 0.67 (molar percentage) | NA | →4)-α-D-Glc*p*-(1→ | →4)-α-D-Glc*p*-(1→ | Anti-inflammation | (69) |
| *Lycium ruthenicum* | LRP3 | Water extraction, DEAE cellulose-52 and Sephadex G-100 chromatography | 75.6 | Rha: Ara: Gal = 1.0: 14.9: 10.4 (molar ratio) | T-Ara*f*-(1→, →2)-Ara*f*-(1→, →5)-Ara*f*-(1→, T-Gal*p*-(1→, →2,4)-Rha*p*-(1→, →3)-Gal*p*-(1→, →6)-Gal*p*-(1→ and →3,6)-Gal*p*-(1→ | →3)-β-D-Gal*p*-(1→ | →5)-β-D-Ara*f*-(1→, →2)-β-D-Ara*f*-(1→, →6)-β-D-Gal*p*-(1→, →3)-β-D-Gal*p*-(1→, →2,4)-α-L-Rha*p*-(1→ and T-α-L-Ara*f*-(1→ at O-6 | Neuroprotection | (64, 65) |
| *Potentilla anserina* | PAP | Water extraction | NA | Glc: Ara: Gal: Rha = 44.5: 6.85: 6.62: 2.52 (mass percentage) | NA | NA | NA | Kidney protection | (59) |
| *Astragalus membranaceus* | APS0; APS1; APS2 | NA | 11.03; 4.72; 2.61 | Glc, Ara, Rha and Gal | NA | NA | NA | Kidney protection | (60) |
| *Nelumbo nucifera* | NNLP-I-I | Water extraction, DEAE-Sepharose Fast Flow and Sepharose 6 FF chromatography | 16.4 | Ara: Rha: Gal: GalA = 1: 1.2: 1.2: 7.1 (molar ratio) | T-Ara*f*-(1→: →5)-Ara*f*-(1→: →3)-Rha*p*-(1→: →2,4)-Rha*p*-(1→: →3)-Gal*p*-(1→: →3,6)-Gal*p*-(1→: T-GalA*p*-(1→: →4)-GalA*p*-(1→ = 7.9: 1.7: 5.7: 5.6: 3.9: 7.2: 2.1: 65.9 (molar ratio) | →4)-α-GalA*p*-(1→ and →2,4)-α-Rha*p*-(1→ | →3)-α-Rha*p*-(1→ and AG-II at C-4 | Gastrointestinal protection | (48) |
| Hemp seed | HSP | Water extraction | 42.1 | Man: Rha: GlcA: GalN: Gal: Xyl: Ara = 6.85: 4.94: 3.85: 22.19: 1.76: 35.26: 25.16 (molar ratio) | NA | NA | NA | Gastrointestinal protection | (126) |
|  | HSP_0.2_ | Water extraction and Q-Sepharose Fast Flow chromatography | 77.4 | Man: Rha: GlcA: Glc: Gal: Ara = 1.68: 6.61: 4.48: 17.52: 40.93: 28.79 (molar ratio) | NA | NA | NA | Gastrointestinal protection | (61) |
| *Aloe vera* | AP | Ethanol precipitation and ammonium sulfate precipitation | 78.8 | NA | NA | →4)-β-Man*p*-(1→ | NA | Gastrointestinal protection | (40) |
| *Platycodon grandiflorus* | PGP-I-I | Water extraction, DEAE-Sepharose Fast Flow and Sepharose 6 FF chromatography | 27.9 | GalA: Ara: Gal: Rha: Glc: Fuc = 43.2: 32.4: 14.6: 8.3: 0.7: 0.1 (molar ratio) | T-Ara*f*-(1→: →5)-Ara*f*-(1→: →3,5)-Ara*f*-(1→: T-Rha*p*-(1→: →2)-Rha*p*-(1→: →3)-Rha*p*-(1→: →2,4)-Rha*p*-(1→: T-Gal*p*-(1→: →4)-Gal*p*-(1→: →3)-Gal*p*-(1→: →6)-Gal*p*-(1→: →3,6)-Gal*p*-(1→: T-GalA*p*-(1→: →4)-GalA*p*-(1→ = 12.9: 5.6: 13.9: 1.3: 4.0: 0.6: 2.4: 2.6: 9.1: 0.9: 0.7: 1.3: 1.1: 42.1 (molar ratio) | HG and RG-I | →5)-α-L-Ara*f*-(1→, AG-I and AG-II | Gastrointestinal protection | (62) |
| *Codonopsis pilosula* | CPP-1 | Water extraction, DEAE-Sepharose Fast Flow and Hiload™ 26/60 Superdex™ 200 prep grade column chromatography | 21.0 | Ara: Rha: Gal: Glc: GalA = 16.7: 9.4: 12.8: 1.1: 58.9 (molar ratio) | T-Ara*f*-(1→: →5)-Ara*f*-(1→: →3,5)-Ara*f*-(1→: T-Rha*p*-(1→: →2)-Rha*p*-(1→: →2,4)-Rha*p*-(1→: T-Gal*p*-(1→: →4)-Gal*p*-(1→: →3)-Gal*p*-(1→: →3,6)-Gal*p*-(1→: T-GalA*p*-(1→: →4)-GalA*p*-(1→ = 4.5: 4.7: 7.5: 1.0: 3.1: 5.3: 5.7: 2.2: 2.3: 2.6: 4.2: 54.7 (molar ratio) | HG | RG-I | Gastrointestinal protection | (55) |
|  | CPSP-1 |  | 13.1 | Ara: Rha: Gal: GalA = 8.9: 9.3: 11.0: 70.1 (molar ratio) | T-Ara*p*-(1→: →5)-Ara*f*-(1→: →3,5)-Ara*f*-(1→: →2)-Rha*p*-(1→: →2,4)-Rha*p*-(1→: T-Gal*p*-(1→: →3)-Gal*p*-(1→: →3,6)-Gal*p*-(1→: →4)-GalA*p*-(1→ = 6.8: 1.1: 1.0: 5.0: 4.3: 4.4: 1.4: 5.1: 70.1 (molar ratio) | HG and RG-I | AG-II |  | (66) |
| *Codonopsis tangshen* | CTP-1 | Water extraction, DEAE-Sepharose Fast Flow and Hiload™ 26/60 Superdex™ 200 prep grade column chromatography | 29.5 | Ara: Rha: Gal: Glc: GalA = 16.7: 9.4: 12.8: 1.1: 58.9 (molar ratio) | T-Ara*f*-(1→: →5)-Ara*f*-(1→: →3,5)-Ara*f*-(1→: T-Rha*p*-(1→: →2)-Rha*p*-(1→: →2,4)-Rha*p*-(1→: T-Gal*p*-(1→: →4)-Gal*p*-(1→: →3)-Gal*p*-(1→: →3,6)-Gal*p*-(1→: T-GalA*p*-(1→: →4)-GalA*p*-(1→ = 2.4: 3.5: 2.5: 0.9: 2.9: 3.9: 4.6: 3.2: 1.8: 2.8: 3.5: 67.5 (molar ratio) | HG | RG-I | Gastrointestinal protection | (55) |
|  | CTSP-1 |  | 23.0 | Ara: Rha: Gal: GalA = 8.2: 11.2: 18.9: 61.3 (molar ratio) | T-Ara*p*-(1→: →5)-Ara*f*-(1→: →3,5)-Ara*f*-(1→: T-Rha*p*-(1→: →2)-Rha*p*-(1→: →3)-Rha*p*-(1→: →2,4)-Rha*p*-(1→: T-Gal*p*-(1→: →4)-Gal*p*-(1→: →3)-Gal*p*-(1→: →6)-Gal*p*-(1→: →3,6)-Gal*p*-(1→: T-GalA*p*-(1→: →4)-GalA*p*-(1→ = 1.6: 5.8: 0.8: 2.2: 3.9: 1.1: 4.0: 4.5: 5.1: 3.1: 1.3: 4.0: 0.6: 60.6 (molar ratio) | HG and RG-I | AG-I and AG-II |  | (66) |
| Corn silk | AHP-2 | Water extraction, H_2_SO_4_ hydrolysis and Sephadex G-25 chromatography | 12.5 | Rha: Ara: Xyl: Man: Gal: Glc = 4.90: 7.25: 2.82: 21.3: 25.1: 38.6 (molar ratio) | NA | NA | NA | Gastrointestinal protection | (87) |
| **Wood plant** | | | | | | | | | |
| Chestnut shell | PCS-2A | Water extraction, DEAE-Sepharose Fast Flow and Superdex-200 chromatography | 34.023 | Rha: Ara: Gal: Glc: Rib: GalA = 0.019: 0.044: 0.059: 0.052: 0.197: 0.628 (molar ratio) | T-Ara*f*-(1→: →5)-Ara*f*-(1→: →3,5)-Ara*f*-(1→: →2,4)-Rha*p*-(1→: →4)-Gal*p*-(1→: →3,4)-Gal*p*-(1→ = 5.67: 10.84: 5.15: 3.45: 62.09: 12.80 (molar ratio) | →4)-α-D-GalA*p*-(1→ and →2,4)-α-L-Rha*p*-(1→ | α-L-Ara*f*-(1→, →5)-α-L-Ara*f*-(1→ and →3,5)-α-L-Ara*f*-(1→) at O-2 | Liver protection | (30) |
| *Smilax china* L. | SCLP | Water extraction, DEAE cellulose-52 and Sephadex G-50 chromatography | 16.8 | GalA: Ara: Gal: Rha = 23.3: 2.1: 1.7: 1.0 (molar ratio) | T-Glc*p*-(1→: →2)-Glc*p*-(1→: →4)-Glc*p*-(1→: →6)-Glc*p*-(1→: →4,6)-Glc*p*-(1→ = 1.3: 1.8: 20.4: 1.0: 1.4 (molar ratio) | →4)-α-D-Glc*p*-(1→ and →2)-α-D-Glc*p*-(1→ | →6)-α-D-Glc*p*-(1→ and α-D-Glc*p*-(1→ at O-6 | Liver protection | (49, 50) |
| *Anoectochilus zhejiangensis* | AZP-1a | Water extraction, DEAE-Sepharose Fast Flow and Sephacryl S-200 HR chromatography | 34.1 | Man: Glc: Gal = 0.111: 0.753: 0.136 (molar ratio) | T-Man*p*-(1→: →4)-Gal*p*-(1→: →4)-Glc*p*-(1→: →4,6)-Glc*p*-(1→ = 0.044: 0.249: 0.662: 0.044 (molar ratio) | →4)-α-D-Glc*p*-(1→ and →4)-β-D-Gal*p*-(1→ | α-D-Man*p*-(1→ at O-6 | Liver protection | (70) |
|  | AZP-1d |  | 4.568 | Glc: Gal = 0.909: 0.091 (molar ratio) | T-Glc*p*-(1→: →4)-Gal*p*-(1→: →4)-Glc*p*-(1→: →4,6)-Glc*p*-(1→ = 0.125: 0.013: 0.736: 0.125 (molar ratio) |  | α-D-Glc*p*-(1→ at O-6 |  |  |
| Acerola | ACPs | Water extraction | 37.07 | Man: Rha: GlcA: Glc: Xyl: Gal: Ara = 2.10: 4.37: 12.48: 7.25: 13.71: 59.81: 0.28 (molar percentage) | NA | NA | NA | Liver protection | (79) |
| Wild jujube | PWJS | Water extraction | NA | Man: Rha: GlcA: GalA: Glc: Xyl: Gal: Ara = 2.03: 3.74: 1.05: 17.64: 38.59: 3.36: 10.44: 23.16 (molar ratio) | NA | NA | NA | Liver protection | (80) |
| *Pinus koraiensis* pine nut | PNP80b-2 | Water extraction, ethanol precipitation, DEAE cellulose-52 and Sephacryl S400 HR chromatography | 23.0 | Gal: Glc: Rha: Xyl: Ara = 16.75: 11.02: 4.25: 1.20: 0.98 (molar ratio) | →2)-Rha*p*-(1→: →4)-Xyl*p*-(1→: T-Gal*p*-(1→: →2)-Gal*p*-(1→: →6)-Glc*p*-(1→: →4)-Glc*p*-(1→: →2,6)-Gal*p*-(1→: →4,6)-Glc*p*-(1→: →2,3,4)-Ara*p*-(1→ = 11.85: 2.88: 17.66: 17.53: 14.92: 3.75: 14.71: 13.69: 3.01 (molar ratio) | NA | NA | Liver protection | (89, 90) |
|  | PNP40c-1 | Water extraction, ethanol precipitation, DEAE cellulose-52 and Sephacryl S400 HR chromatography | 206 | Ara: Rha: Glc = 2.98: 1.00: 0.52 (molar ratio) | →4)-Ara*p*-(1→: →3)-Rha*p*-(1→: →3,4)-Ara*p*-(1→: →6)-Glc*p*-(1→: T-Glc*p*-(1→ = 1.0: 0.4: 0.1: 0.1: 0.1 (molar ratio) | →3,4)-α-L-Ara*p*-(1→, →4)-α-L-Ara*p*-(1→, →3)-α-L-Rha*p*-(1→ and →6)-β-D-Glc*p*-(1→ | β-D-Glc*p*-(1→ at C-4 | Liver protection | (91, 92) |
| *Sonneratia apetala* | SAP-2 | Water extraction and DEAE cellulose-52 chromatography | 97.3 | Rha: Glc: GalA: GlcA: Gal: Ara = 2.89: 1.19: 74.04: 3.55: 1.47: 16.87 (molar ratio) | T-Ara*f*-(1→: →2)-Rha*p*-(1→: T-Glc*p*-(1→: T-GlcA*p*-(1→: T-GalA*p*-(1→: →3)-Gal*p*-(1→: →4)-GalA*p*-(1→: →4)-Glc*p*-(1→: →3,4)-Gal*p*-(1→: →3,4)-GalA*p*-(1→: →2,4)-GalA*p*-(1→: →4,6)-GalA*p*-(1→ = 0.91: 2.39: 0.77: 0.51: 17.11: 0.92: 69.48: 1.90: 1.00: 1.49: 1.72: 1.80 (molar ratio) | NA | NA | Liver protection | (93) |
| *Schisandra chinensis* | SCAP | Water extraction and DEAE-cellulose ion exchange chromatography | NA | GalA: Glc: Gal: Ara: Rha: Man = 53.60: 30.19: 7.25: 4.30: 3.68: 1.36 (mass percentage) | NA | NA | NA | Liver protection | (81, 82) |
|  | SCP | Water extraction |  | Glc: Gal: GalA: Ara: Rha: Man: GlcA = 39.00: 34.80: 11.90: 6.90: 4.80: 1.12: 0.68 |  |  |  |  | (83) |
|  | SCP | Water extraction | 29.16 | Fuc, Ara, Gal, Glc, Xyl, Man, GalA and GlcA | T-Glc*p*-(1→: T-Gal*p*-(1→: →4)-Man*p*-(1→: →4)-Glc*p*-(1→: →3)-Gal*p*-(1→: →6)-Man*p*-(1→: →3,4)-Gal*p*-(1→: →4,6)-Gal*p*-(1→ = 13.51: 2.52: 5.30: 109.86: 1.00: 3.13: 2.59: 7.01 (molar ratio) | NA | NA | Liver protection | (94) |
| *Morinda citrifolia* L. | NFP | Water extraction and ethanol precipitation | 456 | GalA: Gal: Rha: Ara: Glc = 58.42: 4.44: 4.84: 2.16: 21.13 (mass percentage) | T-Ara*f*-(1→: →5)-Ara*f*-(1→: →3,5)-Ara*f*-(1→: T-Glc*p*-(1→: →4)-Glc*p*-(1→: →3,4)-Glc*p*-(1→: →2,4)-Rha*p*-(1→: →2)-Rha*p*-(1→: →4)-Gal*p*-(1→: →3,4)-Gal*p*-(1→: →2,4)-Gal*p*-(1→ = 2.34: 1.19: 1.20: 3.43: 11.92: 2.29: 0.75: 1.77: 72.44: 1.25: 1.42 (molar percentage) | →4)-α-GalA*p*-(1→ | →2)-Rha*p*-(1→, →4)-Gal*p*-(1→ and →5)-Ara*f*-(1→ | Liver protection | (95, 96) |
| Mulberry fruit | MFP-Ⅱ | Water extraction and stepwise ethanol precipitation | 115 | GalA: Glc: Gal: Man: Rha: Xyl: Ara = 3.6: 23.1: 10.9: 2.9: 30.5: 2.7: 26.3 (molar percentage) | T-Rha*p*-(1→: →2)-Rha*p*-(1→: →4)-Gal*p*-(1→: →6)-Gal*p*-(1→: T-Ara*f*-(1→ = 6.9: 20.1: 38.8: 6.4: 27.8 (molar percentage) | NA | NA | Liver protection | (45) |
| Black mulberry fruit | BP1 | Water extraction and stepwise ethanol precipitation | 155.9 | Man: Rha: Glc: Gal: Xyl: Ara: GlcA: GalA = 4.4: 11.1: 2.8: 18.2: 0.2: 17.3: 1.7: 44.4 (molar ratio) | T-Gal*p*-(1→: →4)-Ara*f*-(1→: →2)-Man*p*-(1→: T-Man*p*-(1→: →2)-Glc*p*-(1→ = 67.7: 22.4: 4.2: 2.9: 2.8 (molar ratio) | NA | NA | Liver protection | (46) |
| *Aronia melanocarpa* | AMP | Water extraction and DEAE cellulose-52 chromatography | NA | Fuc: Rha: Ara: Gal: Glc: Xyl: Man: GalA: GlcA: ManA = 0.14: 0.73: 7.14: 10.61: 76.16: 2.31: 1.25: 1.43: 0.16: 0.07 (mass percentage) | NA | NA | NA | Anti-aging | (78) |
| *Taxus chinensis var. mairei* | PTM | Water extraction, DEAE-Sepharose Fast Flow and Sephacryl S-200 chromatography | 3440 | 2,4-di-OMe-Man: Rha: Ara: Xyl: Gal: Man: GalA: GlcA = 2: 5: 24: 9: 3: 46: 1: 10 (molar ratio) | T-Ara*f*-(1→, →2,5)-Ara*f*-(1→, T-Ara*p*-(1→, T-Xyl*p*-(1→, →3)-Xyl*p*-(1→, →4)-Gal*p*-(1→, T-Man*p*-(1→, →3,6)-Man*p*-(1→, T-Glc*p*-(1→, →4)-Glc*p*-(1→, →2)-Rha*p*-(1→ | →3,6)-Man*p*-(1→ | NA | Anti-aging | (73, 74) |
| *Athyrium multidentatum* | AMC | Water extraction | 33.203 | Man: Rha: GlcA: Glc: Gal: Ara: Fuc = 0.077: 0.088: 0.09: 1: 0.375: 0.354: 0.04 (molar ratio) | NA | NA | NA | Anti-aging | (84) |
|  | PS-1 | Water extraction and DEAE cellulose-52 chromatography | 14.561 | Man: Rha: GlcA: Glc: Gal: Ara: Fuc = 0.13: 0.06: 0.01: 1: 0.37: 0.38: 0.03 (molar ratio) | NA | NA | NA | Antioxidant | (85) |
|  | PS-2 |  | 19.783 | Man: Rha: GlcA: Glc: Gal: Ara: Fuc = 0.19: 0.36: 0.63: 0.72: 1: 0.54: 0.29 (molar ratio) |  |  |  |  |  |
|  | PS-3 |  | 4.973 | Man: Rha: GlcA: Glc: Gal: Ara: Fuc = 0.28: 0.37: 0.79: 0.87: 1.32: 1: 0.20 (molar ratio) |  |  |  |  |  |
|  | PS-4 |  | 15.928 | Man: Rha: GlcA: Glc: Gal: Ara: Fuc = 0.20: 0.32: 0.60: 0.64: 1: 0.42: 0.34 (molar ratio) |  |  |  |  |  |
|  | PS-5 |  | 7.046 | Man: Rha: GlcA: Glc: Gal: Ara: Fuc = 0.24: 0.49: 0.32: 1.24: 1: 0.54: 0.24 (molar ratio) |  |  |  |  |  |
| *Opuntia milpa alta* | MAPs | Water extraction and DEAE-Sepharose Fast Flow chromatography | 529 | Ara: Xyl: Gal = 36: 23: 15 (mass percentage) | NA | NA | NA | Anti-diabetic | (127) |
| *Cyclocarya paliurus* | CPP_0.1_ | Water extraction and DEAE cellulose-52 chromatography | 38.4 | Ara: Gal = 0.473: 0.504 (molar ratio) | T-Ara*f*-(1→: →5)-Ara*f*-(1→: T-Gal*p*-(1→: →3)-Gal*p*-(1→: →3,4)-Gal*p*-(1→: →3,6)-Gal*p*-(1→: →3,5,6)-Gal*f* -(1→ = 36: 6: 5: 28: 4: 15: 6 (molar ratio) | →3)-β-D-Gal*p*-(1→ | α-L-Ara*f*-(1→ at O-6 and O-5; β-D-Gal*p*-(1→ and →3,5,6)-β-D-Gal*f*-(1→ at O-4 | Antioxidant | (72) |
|  | CPP_0.05_ |  | 30.160 | Rha: Ara: Glc: Gal = 0.007: 0.233: 0.481: 0.278 (molar ratio) | T-Ara*f*-(1→: T-Glc*p*-(1→: →4)-Glc*p*-(1→: →3)-Gal*p*-(1→: →3,4)-Gal*p*-(1→: →4,6)-Gal*p*-(1→: →3,6)-Gal*p*-(1→: →3,5,6)-Gal*f* -(1→ = 0.200: 0.044: 0.447: 0.062: 0.032: 0.053: 0.109: 0.053 (molar ratio) | →3)-β-D-Gal*p*-(1→ and →4)-α-D-Glc*p*-(1→ | α-L-Ara*f*-(1→ and α-D-Glc*p*-(1→ at O-6, O-5 and O-4 | Antioxidant | (97, 98) |
| *Chimonanthus nitens Oliv* | COP1 | Water extraction and DEAE cellulose-52 chromatography | 18.843 | Ara: Gal: Xyl: Glc = 56.6: 24.9: 11.1: 7.4 (molar ratio) | NA | NA | NA | Antioxidant | (128) |
| *Tetrastigma hemsleyanum* | TTP-1 | Water extraction and DEAE cellulose-52 chromatography | 478.33 | Man: GlcA: GalA: Gal: Ara = 38.91: 14.87: 1.31: 42.81: 2.1 (mass percentage) | NA | NA | NA | Anti-inflammation | (71) |
|  | TVP | Water extraction and DEAE-cellulose 52 chromatography | 64.89 | Man: Rha: GlcA: Glc: Gal: Ara = 35.83: 0.73: 19.15: 1.07: 39.44: 3.77 (molar percentage) | NA | NA | NA | Gastrointestinal protection | (86) |
| *Annona muricata* | ALP | Water extraction | NA | Gal: Glc: Man: Fuc: GalN: GlcN = 64.3: 24.37: 9.81: 0.51: 0.06: 0.93 (mass percentage) | NA | NA | NA | Neuroprotection | (75) |
| *Citrus aurantium* | CALB-3 | Water extraction, Amberlite FPA90-Cl (Cl^−^ form) and Amberlite FPC3500 (H^+^ form), and DEAE-cellulose 52 chromatography | ＞805.0 | Man: Rha: GlcA: GalA: Gal: Ara = 4.2: 4.5: 1.7: 6.1: 45.8: 37.8 (molar percentage) | →6)-Gal*p*-(1→, →4)-GalA*p*-6-OMe-(1→, →3)-Gal*p*-(1→, →3)-Ara*p*-(1→, →3,4)-Gal*p*-(1→ and →6)-Man*p*-(1→ | →3)-α-D-Gal*p*-(1→, →3,4)-α-D-Gal*p*-(1→ and →3)-β-D-Ara*p*-(1→ | →6)-α-D-Gal*p*-(1→, →4)-α-D-GalA*p*-6-OMe-(1→ and →6)-β-D-Man*p*-(1→ at O-3 and/or O-4 | Cardioprotection | (99, 100) |
| **Alga** | | | | | | | | | |
| *Laminaria japonica* | LF | NA | 250 | Man: Rha: Gal: Xyl: Fuc = 2.04: 0.58: 1.04: 3.91: 12.43 (molar ratio) | NA | NA | NA | Liver protection | (101) |
|  | Fucoidan | Water extraction, alkali precipitation and ethanol precipitation | ~7 | Fuc: Gal = 1: 0.24 (molar ratio) | NA | NA | NA | Anti-aging | (102) |
| *Enteromorpha prolifera* | EPP | Water extraction, DEAE cellulose-52 and Sephadex G-100 chromatography | NA | Rha: GlcA: Ara: Fuc: Xyl: Glc = 5.12: 1.32: 3.38: 1.62: 1: 1.03 (molar ratio) | NA | D-GlcUA*p*-α-(1→4)-3-sulfate-L-Rha*p*-β-(1→4)-3-sulfate-L-Rha*p* and D-GlcUA*p*-α-(1→4)-3-sulfate-L-Rha*p*-β-(1→4)-D-Xyl*p*-β-(1→4)-3-sulfate-L-Rha*p* | NA | Liver protection | (31) |
|  | MAP | Enzymatic hydrolysis and ethanol precipitation | 4.929 | Rha: GlcA: Glc: Gal: Xyl = 40.6: 9.3: 38.2: 5.6: 6.3 (molar percentage) | NA | NA | NA | Immunomodulation | (24) |
| *Sargassum fusiforme* | SP2 | Water extraction and CaCl_2_ precipitation | 52.7 | Man: Rha: GlcA: GalA: Glc: Gal: Xyl: Fuc = 3.50: 1.77: 1.11: 2.61: 11.33: 11.51: 12.10: 52.89 (mass percentage) | NA | NA | NA | Anti-aging | (103) |
| *Sargassum kjellmanianum* | SARP | Sephacryl S-300 chromatography | 45.4 | ManA: GulA: GlcA = 76.56: 18.89: 4.55 (mass percentage) | NA | NA | NA | Anti-diabetic | (17) |
| *Hizikia fusiforme* | HFPS-F4 | Enzymatic hydrolysis, ethanol precipitation and DEAE cellulose-52 chromatography | 102.67 | Fuc: Rha: Glc: Man: Ara = 79.20: 2.09: 0.19: 18.13: 0.38: 102.67 (molar ratio) | NA | NA | NA | Antioxidant | (44) |
| **Fungus** | | | | | | | | | |
| *Trametes orientalis* fruiting body | TOP-2 | Ultrasonic-assisted extraction, DEAE cellulose-52 and Sephadex G-100 chromatography | 63 | Gal: Glc: Man: Ara = 5.79: 5.77: 3.45: 1 (molar ratio) | NA | NA | NA | Lung protection | (34, 35) |
| *Sarcodon aspratus* fruiting body | SAFP | Water extraction | NA | Man: Glc: Gal: Fuc = 1.0: 5.16: 4.75: 1.34 (molar ratio) | NA | NA | NA | Lung protection | (109) |
|  |  |  |  |  |  |  |  | Gastrointestinal protection | (110) |
| *Morchella esculenta* fruiting body | FMP-1 | Water extraction, ultrafiltration and Superdex-75 chromatography | 4.7 | Man: Glc: Gal = 1.00: 7.84: 1.24 (molar ratio) | →6)-Man*p*-(1→: T-Glc*p*-(1→: →4)-Glc*p*-(1→: →6)-Gal*p*-(1→: →4,6)-Glc*p*-(1→ = 1.00: 2.18: 4.69: 1.16: 1.34 (molar ratio) | →4)-α-D-Glc*p*-(1→ and →6)-α-D-Gal*p*-(1→ | →6)-β-D-Man*p*-(1→ and T-α-D-Glc*p*-(1→ at O-6 | Lung protection | (32, 33) |
| *Grifola frondosa* fruiting body | GFP | Water extraction, DEAE-Sephadex A-25 and Sephadex G-100 chromatography | 155 | Rha: Xyl: Man: Glc = 1.00: 1.04: 1.11: 6.21 (molar ratio) | →3)-Glc*p*-(1→: T-Glc*p*-(1→: →4)-Man*p*-(1→: →3,4)-Glc*p*-(1→: →3)-Man*p*-(1→: →6)-Rha*p*-(1→: T-Xyl*p*-(1→: →2,4)-Glc*p*-(1→ = 50.67: 3.29: 4.01: 24.62: 4.02: 4.14: 4.34: 4.91 (molar percentage) | →3)-Glc*p*-(1→ and →3,4)-Glc*p*-(1→ | NA | Liver protection | (51, 52) |
| *Pleurotus geesteranus* fruiting body | PFP-1 | Water extraction, DEAE cellulose-52 and Sephadex G-100 chromatography | 15.5 | Fuc: Ara: Gal: Glc: Xyl: Man: Rib= 1.03: 0.18: 21.58: 95.62: 0.48: 13.89: 1.22 (molar ratio) | T-Glc*p*-(1→: T-Man*p*-(1→: T-Gal*p*-(1→: →3)-Glc*p*-(1→: →2)-Man*p*-(1→: →6)-Man*p*-(1→: →6)-Gal*p*-(1→: →4)-Man*p*-(1→: →6)-Glc*p*-(1→: →3,6)-Glc*p*-(1→: →2,6)-Gal*p*-(1→ = 29.70: 0.45: 1.29: 3.16: 0.42: 1.73: 0.86: 1.33: 33.68: 0.28: 27.09 | NA | NA | Liver protection | (107) |
| *Termitomyces albuminosus* mycelium | MPT-W | Water extraction, DEAE cellulose-52 and Sephadex G-100 chromatography | 130 | Xyl: Fuc: Man: Gal: Glc = 0.29: 8.67: 37.89: 35.98: 16.60 (molar ratio) | NA | NA | NA | Liver protection | (111) |
| *Inonotus obliquus* | IOP | Water extraction, DEAE-cellulose ion exchange and Sephadex G-200 chromatography | 42 | Man: Rha: Glc: Gal: Xyl: Ara = 2.2: 1.1: 11.8: 2.8: 2.7: 1.0 (molar ratio) | NA | NA | NA | Liver protection | (108) |
|  |  |  |  |  |  |  |  | Improving reproductive function | (27) |
|  | IOPS | Water extraction, DEAE cellulose-52 and Sephadex G-100 chromatography | 111.9 | NA | NA | NA | NA | Anti-aging | (129) |
| *Cordyceps militaris* fermentation liquid | CEP-I | Ethanol precipitation and Sephadex150 chromatography | 1.206 | Rha: Gal: Glc: GalA: GlcA = 0.130: 47.687: 40.784: 1.795: 0.48 (molar ratio) | NA | →6)-Gal*p*-(1→, →4)-Glc*p*-(1→ and →1,4)-Glc*p*-(6→ | →1)-Rha*f*-(2→ and D-Glc*p*-(1→ | Liver protection | (39) |
| *Amanita caesarea* | ACPS | Water extraction and DEAE cellulose-52 chromatography | 18.620 | Rha, Ara, Xyl, Man, Gal and Glc | NA | NA | NA | Anti-aging | (112) |
|  | ACPS2 | Water extraction, DEAE cellulose-52, Sephacryl S-400 HR and Superdex 200 chromatography | 16.6 | Gal: Glc: Man = 35.40: 31.77: 29.47 (molar ratio) | T-Glc*p*-(1→: T-Man*p*-(1→: →6)-Man*p*-(1→: →2)-Gal*p*-(1→: →3)-Gal*p*-(1→: →6)-Gal*p*-(1→: →3,6)-Gal*p*-(1→: →2,6)-Gal*p*-(1→ = 27.616: 7.614: 31.722: 2.347: 9.477: 10.522: 6.299: 4.404 (molar ratio) | NA | NA |  | (113) |
| *Hericium erinaceus mycelium* | PHEB | Water extraction, stepwise ethanol precipitation, DEAE- Sepharose Fast Flow and Superdex 200 chromatography | 36.1 | Gal, Glc, Man and GlcA | T-Man*p*-(1→: →3)-Glc*p*-(1→: →2)-Man*p*-(1→: →6)-Glc*p*-(1→: →6)-Gal*p*-(1→: →3,6)-Glc*p*-(1→ = 9.74: 32.09: 7.90: 29.15: 11.02: 10.10 (molar ratio) | NA | NA | Anti-aging | (114) |
| *Suillellus luridus* fruiting body | SLPC-1S | Water extraction, DEAE cellulose-52 and Sephadex G-100 chromatography | 9.4 | Gal: Glc: Ara: Man = 44.9: 27.6: 14.7: 12.8 (molar ratio) | T-Gal*p*-(1→: →3)-Gal*p*-(1→: →6)-Gal*p*-(1→: →3,6)-Gal*p*-(1→: →3)-Glc*p*-(1→: →6)-Glc*p*-(1→: →3)-Ara*p*-(1→: →3)-Man*p*-(1→ = 8.5: 17.9: 11.3: 7.8: 13.1: 15.6: 13.7: 11.9 (molar ratio) | →3)-α-D-Gal*p*-(1→, →3)-β-D-Glc*p*-(1→ and →6)-β-D-Glc*p*-(1→ | →3)-β-D-Glc*p*-(1→, →3)-α-L-Ara*p*-(1→, →3)-α-D-Man*p*-(1→ and T-α-D-Gal*p*-(1→ | Anti-diabetic | (115) |
| *Paecilomyces hepiali mycelium* | PHEA | Water extraction, DEAE cellulose-52 and Sephadex G-200 chromatography | 3011.47 | Rha, Gal and Glc | →4)-Rha*p*-(1→, →6)-Glc*p*-(1→, →4)-Glc*p-*(1→ and →6)-Gal*p*-(1→ | NA | NA | Anti-diabetic | (105) |
| *Lentinus edodes mycelium* | LMP | Water extraction, Sephadex G-200 and DEAE cellulose-32 chromatography | 22.7 | Man: Ara: Gal: Xyl: Rha = 1: 0.74: 3.23: 1.18: 10.98 (molar ratio) | NA | NA | NA | Anti-diabetic | (106) |
| *Ganoderm aatrum* fruiting body | PSG-1 | Water extraction and Superdex-G 200 chromatography | 1013 | Glc: Man: Gal: GalA = 4.91: 1: 1.28: 0.71 (molar ratio) | T-Glc*p*-(1→: →3)-Glc*p*-(1→: →6)-Glc*p*-(1→: →3,6)-Gal*p*-(1→: →4,6)-Gal*p*-(1→: →2)-Man*p*-(1→: →4)-Man*p*-(1→: →4)-Gal*p*-(1→: →4)-GalA*p*-(1→ = 3.4: 3.8: 2.7: 2.4: 0.7: 1.5: 0.8: 2.8: 1.4 (molar ratio) | →3)-β-Glc*p*-(1→, →6)-β-Glc*p*-(1→, →4)-α-Gal*p*-(1→, →2)-α-Man*p*-(1→ and →4)-α-Man*p*-(1→ | β-Glc*p*-(1→, →4)-α-Gal*p*-(1→, →4)-α-GalA*p*-(1→, →3)-β-Glc*p*-(1→ and →6)-β-Glc*p*-(1→ at O-3 and O-6 | Gastrointestinal protection | (116, 117) |
| **Animal** | | | | | | | | | |
| *Holothuria leucospilota* | HLP | Enzymatic hydrolysis and ethanol precipitation | 52.80 | GalN: Fuc: GlcA: Gal: Glc: Xyl = 39.08: 35.72: 10.72: 8.43: 4.23: 1.83 (mass ratio) | NA | NA | NA | Liver protection | (118) |
| *Acaudina leucoprocta* | ALP_N_ | Enzymatic hydrolysis and Q Sepharose^TM^ Fast Flow chromatography | 202 | Man: GlcN: Rha: GlcA: GalN: Gal: Fuc = 2.04: 1.30: 3.57: 5.70: 18.73: 15.12: 65.81 (mass ratio) | NA | NA | NA | Antioxidant | (119) |
| *Ostrea talienwhanensis* Crosse | OG | Enzymatic hydrolysis, DEAE cellulose-52 and Sephadex G-100 chromatography | 58 | Glc | T-Glc*p*-(1→: →4)-Glc*p*-(1→: →4,6)-Glc*p*-(1→: →6)-Glc*p*-(1→: →2,4)-Glc*p*-(1→: →3)-Glc*p*-(1→ = 1: 8.103: 1.224: 0.082: 0.096: 0.022 (molar ratio) | NA | NA | Improving reproductive function | (42, 43) |
| **Bacterium** | | | | | | | | | |
| *Bacillus megaterium* | HFC | Ethanol precipitation, DEAE cellulose-52 and Sephadex G-100 chromatography | 128 | Fuc: Glc: Man: Gal: GlcNAc = 41.9: 26.6: 15.8: 12.2: 3.5 (relative percentage) | →2,4)-Gal*p*-(1→: →4)-Gal*p*-(1→: →2,4)-Man*p*-(1→: →4,6)-Man*p*-(1→: →2,4)-Glc*p*-(1→: →4)-Glc*p*-(1→: →3)-Fuc*p*-(1→: →4)-GlcNAc = 8.2: 7.6: 9.7: 8.8: 15.7: 12.9: 31.5: 5.6 (relative percentage) | →4,6)-α-D-Man*p*-(1→, →2,4)-α-D-Man*p*-(1→, →4)-β-D-Glc*p*-(1→, →2,4)-β-D-Glc*p*-(1→ and →4)-β-D-GlcNAc | →2,4)-β-D-Gal*p*-(1→, →4)-β-D-Gal*p*-(1→ and →3)-α-L-Fuc4SO3*p*-(1→ | Lung protection | (37, 38) |
|  | LFC |  | 170 | Gal: Ara: Man: Glc: Fuc: GlcNAc = 37.6: 20.2: 19.3: 14.0: 4.9: 4.0 (relative percentage) | →2,4)-Gal*p*-(1→: →4)-Gal*p*-(1→: →4,6)-Gal*p*-(1→: →4)-Man*p*-(1→: →4,6)-Man*p*-(1→: →2,4)-Glc*p*-(1→: →4,6)-Glc*p*-(1→: →3,4)-Ara*p*-(1→: →3)-Ara*p*-(4→: →1)-Fuc*p*: →1)-GlcNAc = 13.2: 12.3: 11.8: 11.2: 9.4: 8.1: 7.7: 8.4: 8.2: 5.6: 4.1 (relative percentage) | →4,6)-α-D-Man*p*-(1→, →4)-α-D-Man*p*-(1→, →4,6)-β-D-Glc*p*-(1→ and →2,4)-β-D-Glc*p*-(1→ | →1)-β-D-GlcNAc*p*, →1)-α-L-Fuc4SO3*p*, →4)-β-D-Gal*p*(1→, →4,6)- β-D-Gal*p*-(1→, →2,4)-β-D-Gal*p*-(1→, →3,4)-β-L-Ara*p*-(1→ and →3)-β-L-Ara*p*-(1→ | Anti-cancer | (53) |

NA: not available information.
